# VCP downstream metabolite glycerol-3-phosphate (G3P) inhibits CD8^+^T cells function in the HCC microenvironment

**DOI:** 10.1038/s41392-024-02120-8

**Published:** 2025-01-24

**Authors:** Cheng Cheng, Qingrui Zha, Linmao Sun, Tianming Cui, Xinyu Guo, Changjian Xing, Zhengxiang Chen, Changyong Ji, Shuhang Liang, Shengwei Tao, Junhui Chu, Chenghui Wu, Qi Chu, Xuetian Gu, Ning Zhang, Yumin Fu, Shumin Deng, Yitong Zhu, Jiabei Wang, Yao Liu, Lianxin Liu

**Affiliations:** 1 Department of Hepatobiliary Surgery, Centre for Leading Medicine and Advanced Technologies of IHM, The First Affiliated Hospital of USTC, Division of Life Sciences and Medicine, University of Science and Technology of China, Hefei, Anhui 230001 China; 2 Anhui Provincial Key Laboratory of Hepatopancreatobiliary Surgery, Hefei, Anhui 230001 China; 3 Anhui Provincial Clinical Research Center for Hepatobiliary Diseases, Hefei, Anhui 230001 China; 4 Department of Gastrointestinal Surgery, Anhui Province Key Laboratory of Hepatopancreatobiliary Surgery, The First Affiliated Hospital of USTC, Division of Life Sciences and Medicine, University of Science and Technology of China, Hefei, 230001 China

**Keywords:** Cancer microenvironment, Drug development, Immunotherapy, Tumour immunology

## Abstract

CD8^+^T cells within the tumor microenvironment (TME) are often functionally impaired, which limits their ability to mount effective anti-tumor responses. However, the molecular mechanisms behind this dysfunction remain incompletely understood. Here, we identified valosin-containing protein (VCP) as a key regulator of CD8^+^T cells suppression in hepatocellular carcinoma (HCC). Our findings reveal that VCP suppresses the activation, expansion, and cytotoxic capacity of CD8^+^T cells both in vitro and in vivo, significantly contributing to the immunosuppressive nature of the TME. Mechanistically, VCP stabilizes the expression of glycerol-3-phosphate dehydrogenase 1-like protein (GPD1L), leading to the accumulation of glycerol-3-phosphate (G3P), a downstream metabolite of GPD1L. The accumulated G3P diffuses into the TME and directly interacts with SRC-family tyrosine kinase LCK, a critical component of the T-cell receptor (TCR) signaling pathway in CD8^+^T cells. This interaction heightens the phosphorylation of Tyr505, a key inhibitory residue, ultimately reducing LCK activity and impairing downstream TCR signaling. Consequently, CD8^+^T cells lose their functional capacity, diminishing their ability to fight against HCC. Importantly, we demonstrated that targeting VCP in combination with anti-PD1 therapy significantly suppresses HCC tumor growth and restores the anti-tumor function of CD8^+^T cells, suggesting synergistic therapeutic potential. These findings highlight a previously unrecognized mechanism involving VCP and G3P in suppressing T-cell-mediated immunity in the TME, positioning VCP as a promising upstream target for enhancing immunotherapy in HCC.

## Introduction

Globally, hepatocellular carcinoma (HCC) holds a prominent position among cancers, standing as the third major contributor to cancer-related deaths and being highly prevalent.^1^ Chronic liver conditions, notably infections with hepatitis B or C and cirrhosis, frequently lead to development of HCC, the predominant type of liver cancer, thereby rendering its treatment particularly difficult. The prognosis for HCC patients, particularly those diagnosed at advanced stages, remains poor, due to limited therapeutic options and the often late detection of the disease. Over the past years, utilization of immune checkpoint blockade (ICB) has risen as a hopeful approach for treatment of patients diagnosed with advanced hepatocellular carcinoma,^2^ with several immune checkpoint inhibitors showing potential for improving survival outcomes, such as anti-PD1/L1 and anti-CTLA4 antibodies,^3–5^ have aroused attention for their capacity to restore anti-tumor immune responses by disrupting inhibitory signals that hinder immune cells from attacking cancer cells. Despite the success of ICB in various cancers, including durable responses in advanced cases, the efficacy in HCC remains limited, benefiting fewer than 20% of patients.^6–8^ The limited response can be attributed to factors such as the immunosuppressive TME, lower tumor mutational burden, and mechanisms of resistance that are specific to HCC.

TME manifests significant heterogeneity, which plays a critical role in shaping immune response and influencing tumor progression. Among the various immune cells within TME, CD8^+^T cells are considered central to anti-tumor immunity.^9^ In early stages of tumorigenesis, naive CD8^+^T cells undergo antigen and costimulatory signal activation, initiating TCR signal transduction wherein LCK, a Src family tyrosine kinase, serves as a key player in antigen-specific TCR signaling.^10,11^ Proper activation of CD8^+^T cells is vital for mounting an effective immune response against the tumor. However, tumor cells produce immunosuppressive metabolites in the TME, leading to poor activation and impaired function of CD8^+^T cells.^12^ These metabolites, such as lactate, kynurenine, cholesterol, succinate, and D-2-hydroxyglutarate (D-2HG), accumulate within the TME and impair the ability of CD8^+^T cells,^13–17^ leading to immune exhaustion and dysfunction. The consequence is a weakened response against tumors, enabling the malignancy to escape immune detection. In light of these challenges, recent studies have shown that combination therapies, such as combining metabolic modulators like formate with anti-PD1, can promote CD8^+^T cells-mediated tumor control and mouse survival.^18^ This approach helps to overcome metabolic inhibition in CD8^+^T cells, promoting better immune activation and tumor clearance. Although the interaction between CD8^+^T cells and tumor cells in TME is becoming increasingly well understood,^19^ the precise mechanisms remain unclear. Moreover, effective strategies to combine metabolic interventions with immunotherapies to reverse CD8^+^T cell dysfunction are still being explored and require further investigation.^20,21^

The valosin-containing protein (VCP), an extremely preserved member within the AAA class of adenosine triphosphateases (ATPases), is implicated in diverse cellular processes including protein homeostasis regulation, autophagy, chromatin remodeling, transcriptional regulation, and immune signaling.^22,23^ VCP plays a critical role in unfolding ubiquitinated substrate proteins, which are either degraded by the proteasome or deubiquitinated to achieve recycling.^24^ This function is essential for maintaining cellular homeostasis and ensuring the proper functioning of various signaling pathways. Numerous investigations have identified a link between VCP and the advancement of various cancer types, encompassing triple-negative breast carcinoma, colorectal neoplasia, and lung malignancy.^25,26^ Despite its established role in oncogenesis, the specific involvement of VCP in HCC and its impact on the TME remain largely unexplored. Given the crucial character of the TME in shaping cancer progression and response to therapy, understanding VCP’s contribution to this dynamic environment is crucial. Interestingly, recent studies have shown that VCP inhibition can improve the effectiveness of cancer immunotherapy by modulating immune responses.^27,28^ However, the precise mechanisms underlying these effects in the context of HCC remain poorly understood, representing a significant knowledge gap. The biosynthesis of glycerol-3-phosphate (G3P) constitutes an alternative metabolic route to glycolysis, involving the conversion of dihydroxyacetone phosphate (DHAP), a glycolytic intermediate, into G3P, a process facilitated by cytosolic enzymes, namely GPD1 and GPD1L.^29^ G3P serves as a precursor for lipid biosynthesis, supporting the growth and survival of certain cancers, including kidney and ovarian cancer.^30,31^ Beyond its metabolic role, the potential influence of G3P on the immune microenvironment has received limited attention. Understanding how this pathway interacts with immune cells and influences immune evasion or activation in cancers like HCC could provide novel views into the metabolic control of the TME.

In this research, we elucidated the negative relationship between VCP and the infiltration, activation, proliferation, and effector function of CD8^+^T cells within TME, thereby expediting malignant progression in HCC. It was further clarified that VCP facilitated the accumulation of G3P by stabilizing GPD1L, a key enzyme in G3P biosynthesis. This accumulation promoted G3P binding to LCK, an upstream protein in the TCR signaling pathway, thereby inhibiting its activity and leading to CD8^+^T cells dysfunction. This dysfunction highlights a novel mechanism by which VCP mediates immune suppression in the TME. Eventually, we assessed the therapeutic potential of VCP inhibition or Vcp knockout in combination with anti-PD1 therapy in vivo, demonstrating significant improvements in immune responses and tumor control. These findings offer a promising and innovative immunotherapeutic strategy for addressing the challenges of HCC treatment.

## Results

### VCP impairs CD8^+^T cells infiltration, activation and effector function to promote HCC progression

**Fig. 1:**
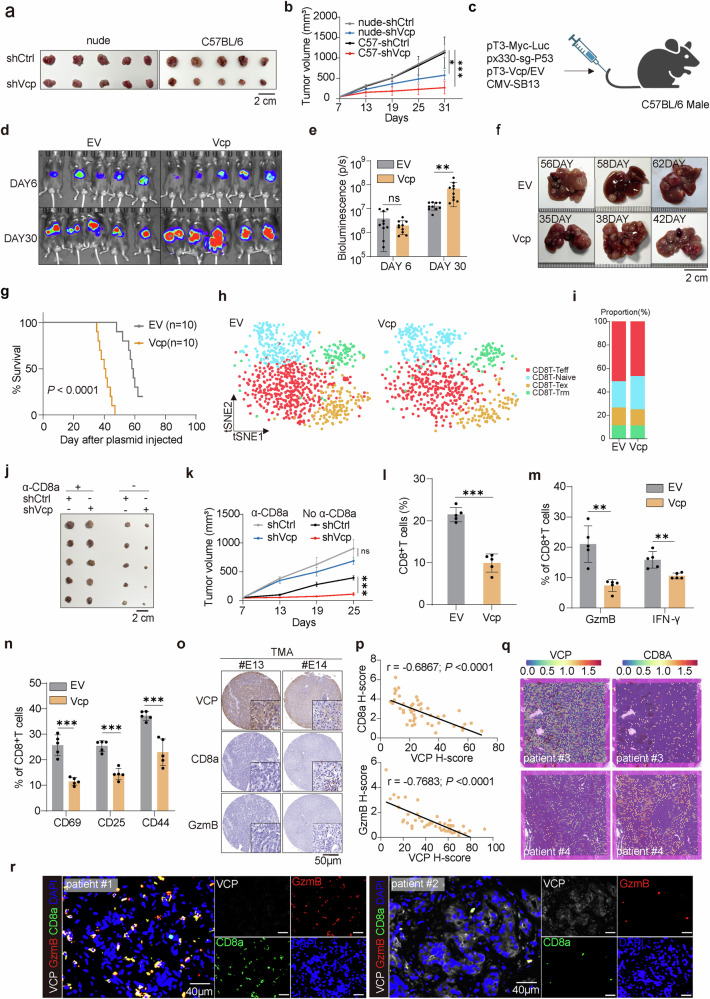
VCP impairs CD8^+^T cells infiltration, activation, and effector function to promote HCC progression. **a**, **b** Representative images of tumor volumes from Hepa1-6-bearing nude and C57BL/6 mice sacrificed on day 31. The average sizes of tumors were measured and plotted (*n* = 5/group). Mean values ± SEM. Two-way ANOVA. **c** Schematic illustrating transposon- and CRISPR-Cas9-based injection of vectors into mice via the tail vein to generate liver tumors resembling human HCC. The transposon-based vector overexpressing Myc can express luciferase. Schematic created with BioRender.com. **d**, **e** Bioluminescence images of C57BL/6 mice at indicated time points after plasmid injection. Bioluminescence signal at indicated time points post plasmid injection in C57BL/6 mice. **f** Representative images of livers from (**d**). **g** Kaplan–Meier survival curve of C57BL/6 mice treated as in (**d**). Log-rank test. **h**, **i** t-SNE analysis of scRNA-seq data of 4 subclusters from CD8^+^T cells. **j**, **k** Representative images of tumor volumes from Hepa1-6-bearing C57BL/6 mice treated with anti-CD8 and sacrificed on day 25 (*n* = 5/group). Mean values ± SEM. Two-way ANOVA. **l**–**n** The percentage of CD8^+^T cells (**l**), cytokines production (Granzyme B, IFN-γ) (**m**), and activation indicators (CD69, CD25, CD44) (**n**) in spontaneous tumor were measured by flow cytometry (*n* = 5/group). **o** Representative tissue microarray (TMA) images for VCP, CD8a, and GzmB immunohistochemistry in 90 HCC patients. **p** The IHC staining was scored, and correlation analyses were performed (*n* = 90 patients). The Pearson correlation test was used. **q** Signature score of VCP and CD8A expression in patient #3 and #4. **r** Patients clinical samples were stained with multiple immunofluorescence for DAPI, CD8a, GzmB, and VCP. Data are presented as mean values ± SD. Statistical significance was determined using two-sided t-tests, *P < 0.05, **P < 0.01, and ***P < 0.001

To ascertain the involvement of VCP in tumor immune responses, we inoculated Hepa1-6 cells depleted of Vcp or control cells into both nude mice (immunodeficient) and C57BL/6 mice (immunocompetent) (Supplementary Fig. 1a–c). Interestingly, both nude and C57 mice injected with depleted Vcp or control cells exhibited changes in tumor growth, but in the immunocompetent model, Vcp depletion suppressed tumor growth more significantly (Fig. 1a, b). This indicated that although VCP itself was oncogenic, in the immune environment, the different tumorigenicity was mostly attributed to immune surveillance.

A previously described genetically modified mouse model of HCC can better mimic the HCC immune microenvironment,^32^ so we developed a murine model of HCC by utilizing a system that combines p53 knockout with Myc overexpression specifically in hepatocytes. Specifically, the plasmids were injected into male mice (Fig. 1c), and the effect of Vcp on HCC progression was regularly monitored. Bioluminescence imaging revealed equivalent luciferase expression in the liver on day 6, but by day 30, luciferase expression was notably higher in the Vcp overexpression group compared to controls (Fig. 1d, e). Moreover, the Vcp overexpression group showed earlier tumorigenesis, larger tumor volume, and shorter survival (Fig. 1f, g and Supplementary Fig. 1b, c). Utilizing single-cell RNA sequencing in combination with t-SNE analysis, we delineated 13 subclusters within the total cell population, with T cells showing distinct reduction in Vcp overexpression group (Supplementary Fig. 1d, e). Among T cells, CD8^+^T cells, the largest subset, were pivotal in immunity (Supplementary Fig. 1f, g), thus we focused our investigation on CD8^+^T cells. Further sub-division of CD8^+^T cells into 4 subsets revealed a reduction in effector CD8^+^T cells in Vcp overexpression group (Fig. 1h, i and Supplementary Fig. 1h). Significantly, upon immunodepletion of CD8^+^T cells, development suppression of tumors with Vcp depletion in immunocompetent mice was nearly abolished (Fig. 1j, k and Supplementary Fig. 1i), underscoring the vital importance of effector CD8^+^T cells in VCP-mediated tumor growth.

Subsequently, we found diminished level of CD8^+^T cells in Vcp-overexpressing tumor, with these CD8^+^T cells exhibiting visibly lower levels of cytokines and activation markers CD69, CD25, CD44^33^ compared to controls (Fig. 1l–n and Supplementary Fig. 2a–e). Consistent outcomes were obtained in the subcutaneous tumor model of HCC (Supplementary Fig. 2f–l). Analysis of a tissue microarray (TMA) containing 90 HCC specimens through immunohistochemistry uncovered a negative relationship between VCP expression and the presence of CD8^+^T cells, along with the expression of the cytokine Granzyme B (Fig. 1o, p). Spatial transcriptome mapping of tumor tissue from clinical patients indicated reduced CD8^+^T cell infiltration in regions exhibiting high VCP expression, contrasting with increased infiltration in regions with low VCP expression (Fig. 1q). Consistent results were received through multiple immunofluorescence staining analyses of clinical patient samples (Fig. 1r). In addition, we evaluated the clinical significance of VCP expression on the survival of HCC patients using the TCGA cohort. It was observed that in contrast to HCC patients exhibiting lower VCP expression, those with higher VCP expression had a less favorable prognosis. Specifically, both the overall survival (OS) and disease-free survival (DFS) were shorter in the latter group (Supplementary Fig. 2m, n). Collectively, the findings indicate that VCP in tumors hinders the anti-tumor capabilities of effector CD8^+^T cells within TME, thereby facilitating the HCC progression.

### VCP inhibits CD8^+^T cells function via metabolite-mediated mechanism

**Fig. 2:**
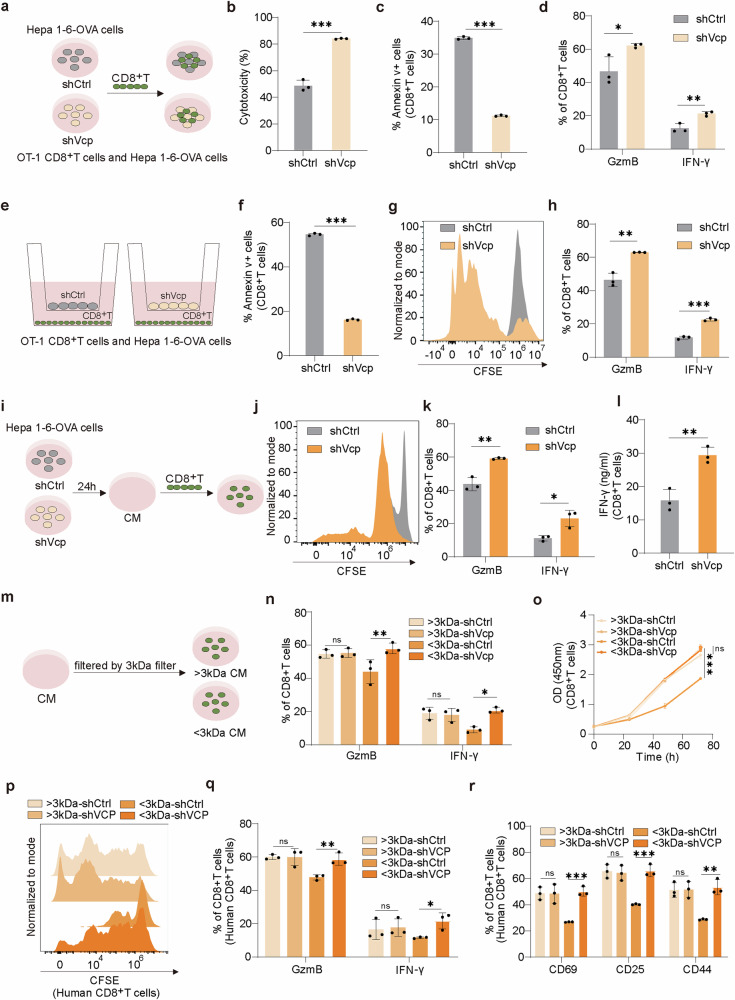
VCP inhibits CD8^+^T cells function via metabolite-mediated mechanism. **a** OT-1 CD8^+^T cells co-cultured with shCtrl or shVcp Hepa1-6-OVA cells. **b** Cytotoxicity assessed by measuring lactate dehydrogenase (LDHA) release from Hepa1-6-OVA cells (*n* = 3). **c** The percentage of Annexin v^+^ CD8^+^T cells co-cultured with shCtrl or shVcp Hepa1-6-OVA cells was determined by flow cytometry (*n* = 3). **d** The percentage of cytokines produced by CD8^+^T cells co-cultured with tumor cells was measured by flow cytometry (*n* = 3). **e** Schematic diagram showing OT-1 CD8^+^T cells co-cultured with shCtrl or shVcp Hepa1-6-OVA cells using a Transwell system. **f** The percentage of Annexin v^+^ CD8^+^T cells in Transwell system was determined by flow cytometry (*n* = 3). **g** The proliferation of CD8^+^T cells in Transwell system was measured by CFSE assay. **h** The percentage of cytokines produced by CD8^+^T cells in Transwell system was measured by flow cytometry (*n* = 3). **i** Schematic diagram showing CD8^+^T cells cultured with conditioned medium (CM) produced by shCtrl or shVcp Hepa1-6-OVA cells. **j** The proliferation of CD8^+^T cells cultured with CM produced by shCtrl or shVcp Hepa1-6-OVA cells was measured by CFSE assay. **k** The percentage of cytokines produced by CD8^+^T cells cultured with CM produced by shCtrl or shVcp Hepa1-6-OVA cells was measured by flow cytometry (*n* = 3). **l** Secreted IFN-γ levels by CD8^+^T cells cultured with CM produced by shCtrl or shVcp Hepa1-6-OVA cells (*n* = 3). **m** Schematic diagram showing CD8^+^T cells cultured with >/<3 kDa CM produced by shCtrl or shVcp Hepa1-6-OVA cells. **n** The percentage of cytokines produced by CD8^+^T cells cultured with >/<3 kDa CM produced by shCtrl or shVcp Hepa1-6-OVA cells was measured by flow cytometry (*n* = 3). **o** The proliferation of CD8^+^T cells cultured with >/<3 kDa CM produced by shCtrl or shVcp Hepa1-6-OVA cells was measured by CCK8 assay (*n* = 3). Two-way ANOVA. **p** The proliferation of human CD8^+^T cells cultured with >/<3 kDa CM produced by shCtrl or shVCP HCCLM3 cells was measured by CFSE assay. **q** The percentage of cytokines produced by human CD8^+^T cells cultured with >/<3 kDa CM produced by shCtrl or shVCP HCCLM3 cells was measured by flow cytometry (*n* = 3). **r** The expression of activation indicators in human CD8^+^T cells cultured with >/<3 kDa CM produced by shCtrl or shVCP HCCLM3 cells was determined by flow cytometry (*n* = 3). Data are presented as mean values ± SD. Statistical significance was determined using two-sided t-tests, *P < 0.05, **P < 0.01, and ***P < 0.001

In order to clarify the function mechanism of VCP, we conducted co-culture of CD8^+^T cells and ovalbumin-expressing Hepa 1–6 cells (Hepa 1-6-OVA) (Fig. 2a). Deletion of Vcp in tumor cells augmented the cytotoxicity of CD8^+^T cells (Fig. 2b), induced apoptosis in tumor cells (Supplementary Fig. 3a), lessened CD8^+^T cell apoptosis (Fig. 2c and Supplementary Fig. 3b), and upregulated the level of cytokines GzmB and IFN-γ (Fig. 2d and Supplementary Fig. 3c, d), consistent with our in vivo observations. After transfer from lymph nodes to the tumor, CD8^+^T cells recognize antigens presented by tumor and exert killing effects.^34^ To determine whether VCP impacts antigen presentation, we conducted non-contact co-culture experiments using a Transwell system (Fig. 2e). Similarly, Vcp depletion in tumor cells increased tumor cells apoptosis (Supplementary Fig. 3e), reduced apoptosis of CD8^+^T cells (Fig. 2f and Supplementary Fig. 3f), promoted proliferation of CD8^+^T cells (Fig. 2g and Supplementary Fig. 3g), as well as cytokines expression (Fig. 2h), which ruled out the impact of VCP on tumor antigen presentation.

To further investigate the mechanism of VCP’s effect, we isolated CD8^+^T cells and cultured them in conditioned medium (CM), which was derived from a 24-h incubation of tumor cells (Fig. 2i). Surprisingly, CM produced by shVcp Hepa1-6-OVA cells promoted the CD8^+^T cells proliferation (Fig. 2j), as well as cytokines level (Fig. 2k, l), suggesting that VCP suppressed CD8^+^T cells functionality through some secretions. To elucidate the characteristics of the secretions, CM was fractionated into >3 kDa (protein-enriched) and <3 kDa (metabolite enriched) fractions by a 3 kDa cutoff centrifugal filter (Fig. 2m). Metabolites (<3 kDa enriched fraction), rather than proteins, contribute to VCP mediated inhibition of CD8^+^T cells function, reduced cytokines expression (Fig. 2n and Supplementary Fig. 3h), and inhibited proliferation of CD8^+^T cells (Fig. 2o) resulting in increased apoptosis (Supplementary Fig. 3i). These findings were corroborated using human CD8^+^T cells, where VCP suppressed the growth of human CD8^+^T cells (Fig. 2p and Supplementary Fig. 3j), increased CD8^+^T cells apoptosis (Supplementary Fig. 3k), reduced cytokines production (Fig. 2q and Supplementary Fig. 3l, m), and dampened the level of activation indicators CD69, CD25, CD44 (Fig. 2r and Supplementary Fig. 3n) via metabolites components.

### VCP acts through downstream metabolite G3P

**Fig. 3:**
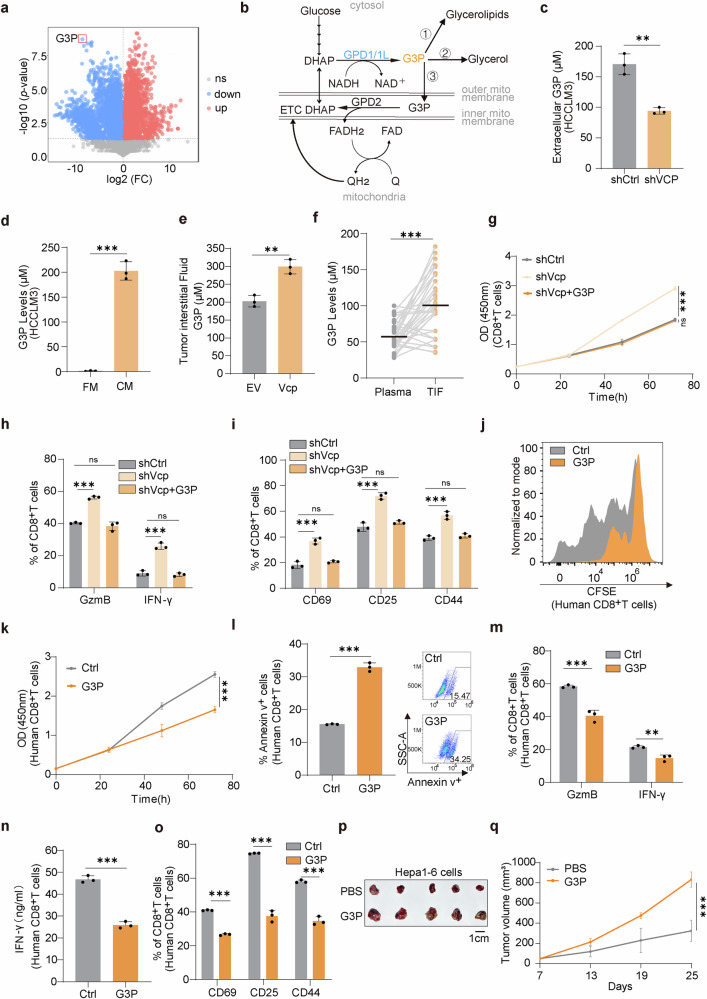
VCP acts through downstream metabolite G3P. **a** Volcano plot representing metabolite changes in conditional medium from shVCP HCCLM3 cells compared with Control. **b** Schematic of G3P metabolism. **c** Extracellular G3P levels in shCtrl or shVCP HCCLM3 cells (*n* = 3). **d** G3P levels in fresh medium (FM) and conditional medium (CM) from HCCLM3 cells (*n* = 3). **e** G3P levels in interstitial fluid of spontaneous tumors (*n* = 3). **f** G3P levels in tumor interstitial fluid (TIF) and plasma from HCC patients (*n* = 31). **g** The proliferation of CD8^+^T cells co-cultured with shCtrl or shVcp Hepa1-6-OVA cells or treated with G3P using a Transwell system was measured by CCK8 assay (*n* = 3). Two-way ANOVA. **h**, **i** The percentage of cytokines production (**h**) and activation indicators (**i**) of CD8^+^T cells treated as in (**g**) were measured by flow cytometry (*n* = 3). **j** The proliferation of human CD8^+^T cells treated with G3P was measured by CFSE assay. **k** The proliferation of human CD8^+^T cells treated with G3P was measured by CCK8 assay (*n* = 3). Two-way ANOVA. **l** The percentage of Annexin v^+^ human CD8^+^T cells treated with G3P was determined by flow cytometry (*n* = 3). **m** The percentage of cytokines produced by human CD8^+^T cells treated with G3P was measured by flow cytometry (*n* = 3). **n** Secreted IFN-γ levels by human CD8^+^T cells treated with G3P. Analysis was performed after 24 h (*n* = 3). **o** The expression of activation indicators in human CD8^+^T cells treated with G3P was determined by flow cytometry (*n* = 3). **p**, **q** Representative images of tumor volumes from Hepa1-6-bearing C57BL/6 mice after intratumoral injection of PBS/G3P and sacrificed on day 25 (*n* = 5/group). Mean values ± SEM. Two-way ANOVA. Data are presented as mean values ± SD. Statistical significance was determined using two-sided t-tests, *P < 0.05, **P < 0.01, and ***P < 0.001

Considering that tumor VCP acts through certain metabolites, we conducted untargeted metabolomics analysis on conditioned medium from VCP-depleted HCCLM3 cells and control cells after 24 h of culture. Although most of the nutrients detected were lower in the medium of VCP depleted HCCLM3 cells, the levels of glycerol-3-phosphate (G3P) changed most significantly (Fig. 3a). G3P serves as a central node, linking lipid synthesis, oxidative phosphorylation, and glycolysis (Fig. 3b). Next we validated the metabolomics results, G3P levels decreased both inside and outside the cells of human and mouse origin in VCP depleted cells (Fig. 3c and Supplementary Fig. 4a–c), and also increased markedly in conditioned medium produced by tumor cells (Fig. 3d and Supplementary Fig. 4d). To ascertain whether G3P displays similar changes in vivo, we isolated tumor interstitial fluid (TIF) from subcutaneous tumors and spontaneous tumor models, finding reduced G3P levels in Vcp-depleted subcutaneous tumors and elevated levels in Vcp-overexpressing spontaneous tumors (Fig. 3e and Supplementary Fig. 4e). In addition, G3P concentrations in TIF isolated from HCC patient tissues were higher than those in matched plasma (Fig. 3f).

To determine the optimal concentration of G3P, gradient concentrations of G3P were added to CD8^+^T cells, and it turned out that the intracellular G3P levels were also increased in a gradient manner (Supplementary Fig. 4f). Notably, the inclusion of G3P, potentially up to 400 µM, was enough to suppress CD8^+^T cell activation (Supplementary Fig. 4g–i). The combination of CD3 and CD28 antibodies stimulates T cells, simulating the dual signaling effect of T cell activation in vivo.^35^ When antigen was absent, G3P itself could not affect CD8^+^T cells activation (Supplementary Fig. 4j–l).

To confirm the role of G3P as a downstream metabolite of VCP, we utilized a Transwell system for non-contact co-culture, wherein G3P supplementation counteracted the pro-proliferation (Fig. 3g), cytokine expression (Fig. 3h), and activation effects (Fig. 3i) of Vcp deletion on CD8^+^T cells. Consistent findings were obtained as we further assessed the direct effects of G3P. G3P suppressed the proliferation of human CD8^+^T cells (Fig. 3j, k), led to apoptosis (Fig. 3l), and suppressed the expression of cytokines (Fig. 3m, n) and activation markers (Fig. 3o). Similar conclusions were drawn from experiments conducted using mouse CD8^+^T cells (Supplementary Fig. 4m–r). Intratumoral injection of G3P in Hepa1-6 tumor-bearing mice also promoted tumor growth (Fig. 3p, q). The evidence implies that VCP’s inhibition of CD8^+^T cells is mediated through downstream metabolite G3P.

### VCP maintains GPD1L stability to accumulate G3P in HCC

**Fig. 4:**
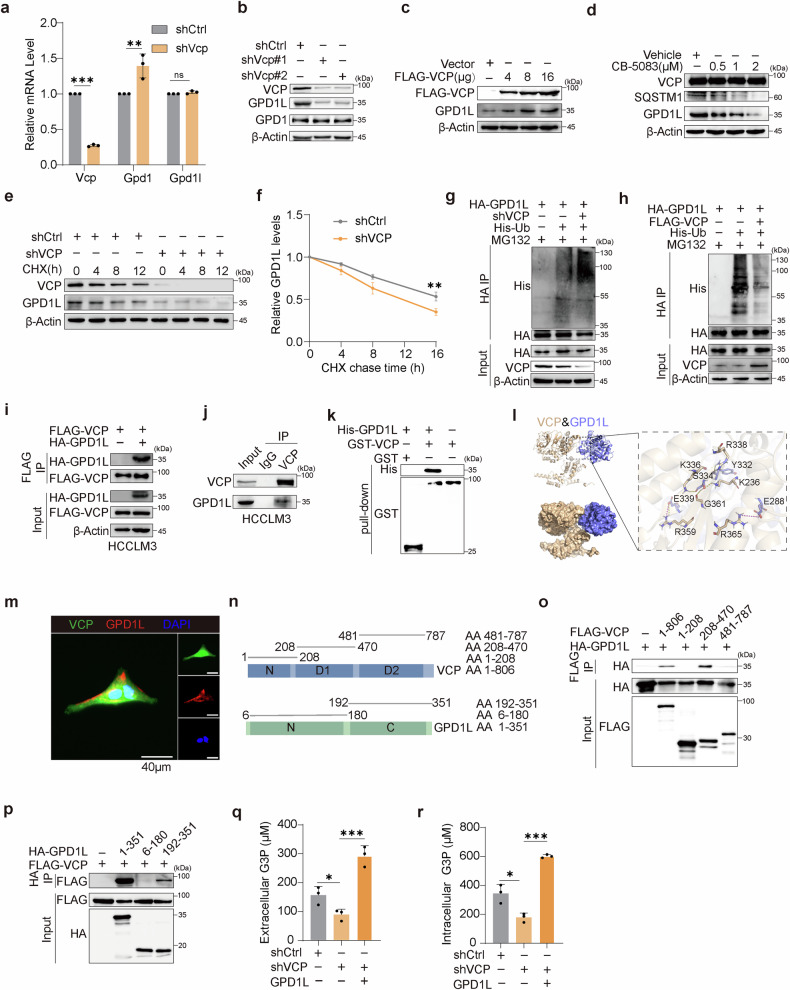
VCP maintains GPD1L stability to accumulate G3P in HCC. **a** qPCR analysis assessing expression of Vcp, Gpd1, and Gpd1l in Hepa1-6 cells stably expressing shCtrl or shVcp (*n* = 3). **b** Immunoblot analysis of the indicated proteins in Hepa1-6 cells stably expressing shCtrl or shVcp. **c** Immunoblot analysis of the indicated proteins in HCCLM3 cells transfected with increasing amounts of FLAG-VCP. **d** Immunoblot analysis of the indicated proteins in HCCLM3 cells treated with gradient concentrations of CB-5083 or Vehicle for 24 h. **e**, **f** Immunoblot analysis of the indicated proteins in shCtrl or shVCP HCCLM3 cells treated with or without 100 μg/mL CHX for the indicated times. Relative GPD1L protein levels (GPD1L:β-Actin) are shown in (**f**). **g**, **h** Immunoblot analysis of HA beads pull-down products and input derived from HCCLM3 cells treated with 10 μM MG132 for 8 h and co-transfected with the indicated plasmids for 48 h. **i** Immunoblot analysis of FLAG beads pull-down products and input derived from HCCLM3 cells co-transfected with the indicated plasmids for 48 h. **j** HCCLM3 cell lysates were subject to immunoprecipitation with control IgG, anti-VCP antibodies. The immunoprecipitates were then blotted. **k** GST pull-down assay was performed by mixing recombinant GST-VCP with pre-immunoprecipitated His-GPD1L protein. **l** The interaction between VCP and GPD1L was speculated using three-dimensional structures. **m** Immunofluorescence analysis to detect colocalization of VCP and GPD1L in HCCLM3 cells. **n** Schematic of VCP and GPD1L structures. **o** Immunoblot analysis of FLAG beads pull-down products and input derived from 293T cells transfected with HA-GPD1L and FLAG-tagged indicated constructs. **p** Immunoblot analysis of HA beads pull-down products and input derived from 293T cells transfected with FLAG-VCP and HA-tagged indicated constructs. **q** Extracellular G3P levels in shCtrl and shVCP HCCLM3 cells transfected with or without HA-GPD1L for 48 h (*n* = 3). **r** Intracellular G3P levels in shCtrl or shGPD1L HCCLM3 cells transfected with or without HA-GPD1L for 48 h (*n* = 3). Data are presented as mean values ± SD. Statistical significance was determined using two-sided t-tests, *P < 0.05, **P < 0.01, and ***P < 0.001

Given that GPD1 and GPD1L serve as upstream kinases of G3P, we sought to investigate whether VCP acts through both kinases. RT-qPCR analysis showed that deletion of Vcp upregulated the mRNA level of Gpd1 but not Gpd1l (Fig. 4a). Protein detection demonstrated that Vcp deletion had no effect on GPD1 level, but reduced GPD1L protein level (Fig. 4b). In addition, we measured the G3P levels in GPD1 and GPD1L knockdown cells and found that GPD1L knockdown significantly reduced both intracellular and extracellular G3P levels, whereas GPD1 knockdown had no effect on G3P levels (Supplementary Fig. 5a–c). This indicates that G3P in HCC mainly depends on GPD1L expression. Therefore, we subsequently focused our research on GPD1L. Deletion of VCP in human HCC cell lines led to reduced GPD1L levels (Supplementary Fig. 5d), while ectopic expression of VCP dose-dependently increased GPD1L expression (Fig. 4c). To serve as a positive control, treatment of HCCLM3 cells with varying concentrations of the VCP enzyme activity inhibitors CB-5083 and NMS873 led to a reduction of SQSTM1, a known VCP substrate.^36^ Similarly, this also caused a dose-dependent decrease in GPD1L protein expression (Fig. 4d and Supplementary Fig. 5e). The results suggested that regulation of GPD1L by VCP was post-transcriptional. To further bolster the view, the cells were exposed to cycloheximide (CHX) treatment, a protein synthesis inhibitor, revealing that GPD1L’s half-life decreased in VCP depleted cells but increased in those overexpressing VCP (Fig. 4e, f and Supplementary Fig. 5f, g). These data, taken together, suggest that VCP functions as a stabilizer for GPD1L.

Two essential cellular degradation systems in eukaryotic cells are ubiquitin-proteasome pathway and autophagy.^37^ To examine the degradation pathway of GPD1L, cells underwent treatment with the MG132 and chloroquine (CQ) for specified durations. The immunoblotting assay demonstrated that the expression of GPD1L was enhanced by MG132 treatment (Supplementary Fig. 5h). In MG132 treated cells, downregulation of VCP increased GPD1L ubiquitination (Fig. 4g), while ectopic expression of VCP decreased GPD1L ubiquitination (Fig. 4h), indicating VCP-mediated stabilization of GPD1L via the ubiquitin-proteasome pathway. In order to clarify the specific regulatory sites of VCP on GPD1L, we predicted 5 ubiquitination sites of GPD1L from the GPS-Uber database^38^ (Supplementary Fig. 5i), and constructed mutants of these sites for validation. After the K226 site mutation, the ubiquitination level of GPD1L significantly decreased (Supplementary Fig. 5j), indicating that VCP mediates the ubiquitination of GPD1L at the K226 site.

To further verify whether VCP directly interacts with GPD1L, HCCLM3 and HEK293T cells were transfected with FLAG-VCP, HA-GPD1L, or both combinations. Reciprocal immunoprecipitation was conducted reciprocally, employing antibodies specific to FLAG or HA tags. Immunoblot analysis showed that Flag-VCP and HA-GPD1L interacted only when both were co-expressed (Fig. 4i and Supplementary Fig. 5k). Consistently, the same results were received when HA-VCP and FLAG-GPD1L plasmids used for the above transfection (Supplementary Fig. 5l, m). Co-immunoprecipitation of endogenous VCP and GPD1L proteins further supported their interaction (Fig. 4j and Supplementary Fig. 5n). Furthermore, GST pull-down assays proved direct binding between purified GST-VCP fusion protein and His-GPD1L protein (Fig. 4k). We also performed molecular docking simulations to model the binding between VCP and GPD1L (Fig. 4l). Immunofluorescence staining certified that VCP partially co-localized with GPD1L (Fig. 4m and Supplementary Fig. 5o). Further, we found that the D1 domain of VCP (Δ208-470 aa) was sufficient for binding to GPD1L, which interacted with VCP via its Δ192-351 aa fragment (Fig. 4n–p).

Subsequently, intracellular and extracellular G3P levels were assessed, revealing that overexpression of GPD1L in VCP-depleted cells significantly increased both intracellular and extracellular G3P levels (Fig. 4q, r and Supplementary Fig. 5p), confirming that VCP promotes G3P accumulation by stabilizing GPD1L expression. In addition, we assessed T cell function and found that restoring Gpd1l expression indeed reversed the Vcp depletion-induced cytotoxic function of T cells (Supplementary Fig. 5q). Furthermore, we measured G3P levels, and consistent with the previous results in Fig. 4q, GPD1L overexpression promoted G3P accumulation (Supplementary Fig. 5r). These results suggest that VCP mediates T cell inactivation by promoting G3P accumulation through downstream GPD1L.

### G3P inhibits CD8^+^T cells through LCK signaling

**Fig. 5:**
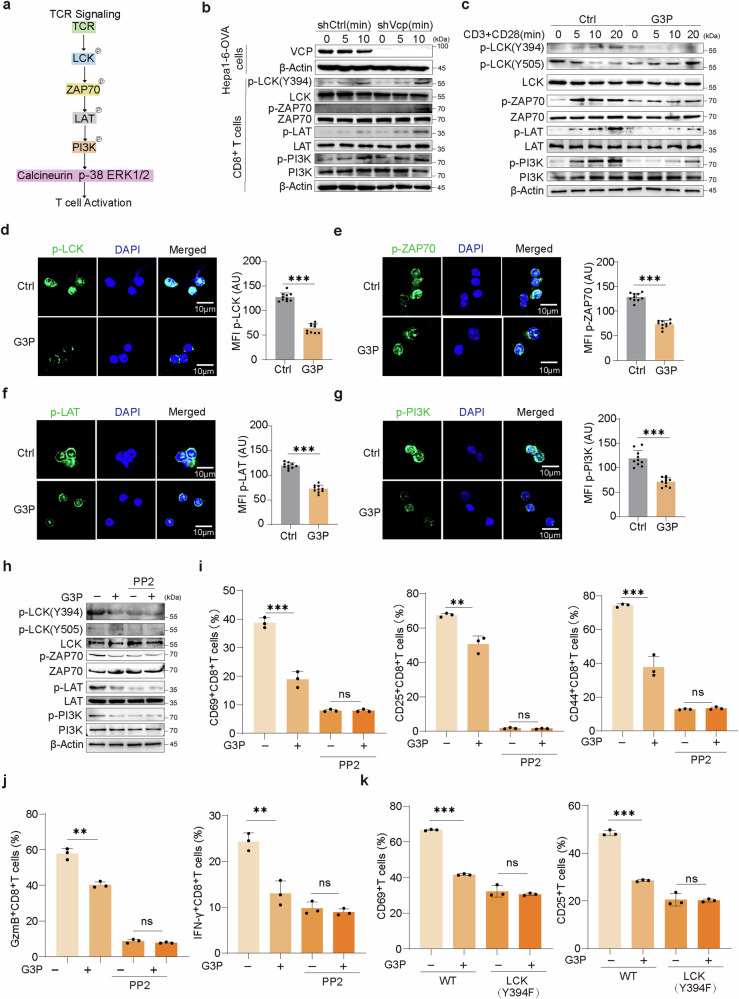
G3P inhibits CD8^+^T cells through LCK signaling. **a** The TCR signaling pathway. **b** CD8^+^T cells co-cultured with shCtrl or shVcp Hepa1-6-OVA cells were activated for 5 or 10 min or unstimulated (0). Cell lysates were analyzed by western blot (WB). **c** Mouse naive CD8^+^T cells treated with G3P or untreated (Ctrl) were activated for the indicated times. Cell lysates were analyzed by western blot (WB). **d**–**g** Left, mouse naive CD8^+^T cells treated with G3P or untreated (Ctrl) followed by stimulation were subjected to immunostaining and confocal microscopic imaging. Right, quantification of the mean fluorescence intensity. **h** Immunoblot analysis of the indicated proteins in mouse CD8^+^T cells treated with G3P and/or PP2 during stimulation. **i** The expression of activation indicators in mouse CD8^+^T cells treated with G3P and/or PP2 was determined by flow cytometry (*n* = 3). **j** The percentage of cytokines produced by mouse CD8^+^T cells treated with G3P and/or PP2 was measured by flow cytometry (*n* = 3). **k** Jurkat T cells expressing WT–LCK or the LCK(Y394F) mutant were treated with G3P or untreated. The expression of activation indicators was determined by flow cytometry (*n* = 3). Data are presented as mean values ± SD. Statistical significance was determined using two-sided t-tests, *P < 0.05, **P < 0.01, and ***P < 0.001

Previous conclusions have shown that VCP suppressed the expression of CD8^+^T cells activation indicators and cytokines (Figs. 1 and 2). The Starting Point of T cells signaling pathway is activation of T cell antigen receptor (TCR). The TCR signaling pathway is primarily controlled at three crucial points: LCK, ZAP70, LAT, to activate downstream signaling pathways (Fig. 5a), which is important for T cell activation and effector function.^39^ Upon co-culture with Hepa1-6-OVA cells, a notable reduction in TCR signaling activation was manifested by CD8^+^T cells, along with reduced phosphorylation of critical signaling pathway components, such as LCK (at Y394) and its downstream proteins, including ZAP70, LAT, and PI3K, but after Vcp knockdown, TCR signaling activation was enhanced (Fig. 5b), suggesting tumor VCP may inhibit CD8^+^T cells by hindering TCR signaling.

As G3P serves as the downstream metabolite of VCP, we investigated its impact on CD8^+^T cell signaling. Addition of G3P to CD8^+^T cells resulted in decreased phosphorylation of LCK (Y394) and its downstream targets, including ZAP70, LAT, and PI3K (Fig. 5c). In addition, G3P impaired the activation of human Jurkat T lymphocyte cells in a dose-dependent approach (Supplementary Fig. 6a). Further by confocal imaging, we observed that G3P treatment diminished microclusters of phosphorylated TCR signaling in CD8^+^T cells (Fig. 5d–g), similar conclusion was gained in Jurkat cells (Supplementary Fig. 6b–e). The outcomes uncovered a novel function of G3P in modulating TCR signaling, which had not been previously recognized. Supplementation with the LCK inhibitor PP2 completely blocked the dephosphorylation of TCR signaling proteins induced by G3P (Fig. 5h). Remarkably, although G3P impaired CD8^+^T cells activation and effector function, PP2 supplementation greatly weakened the inhibitory effect of G3P (Fig. 5i, j and Supplementary Fig. 6f, g), as also verified in Jurkat cells (Supplementary Fig. 6h). Conformably, the expression of LCK (Y394F) led to reduced activation and effector function in Jurkat cells, while also decreasing their sensitivity to G3P (Fig. 5k and Supplementary Fig. 6i, j). The outcomes suggest that suppression of LCK signaling via G3P, CD8^+^T cells activation and effector function can be inhibited.

### G3P directly interacts with LCK and suppresses its activity

**Fig. 6:**
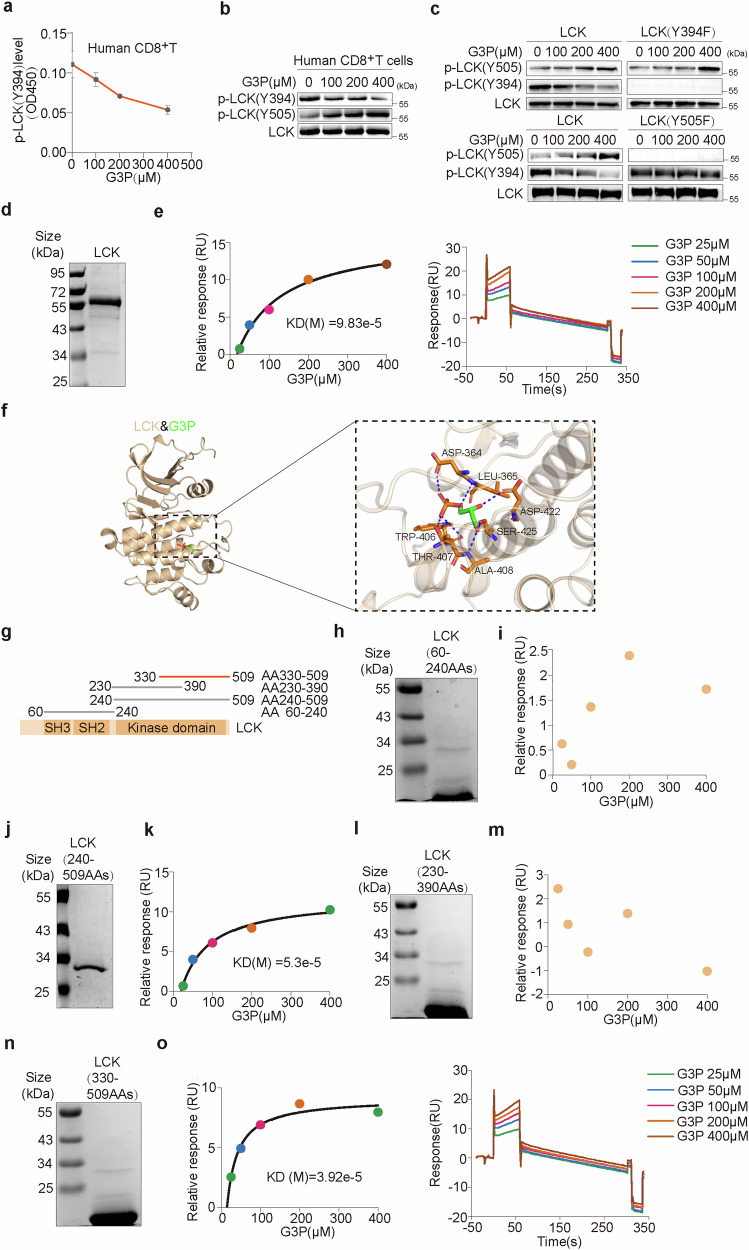
G3P directly interacts with LCK and suppresses its activity. **a** The p-LCK(Y394) levels of human CD8^+^T cells treated with gradient concentrations of G3P. **b** Immunoblot analysis of the indicated proteins in human CD8^+^T cells treated with gradient concentrations of G3P. **c** In vitro kinase assay using purified LCK and its mutants incubated with ATP and increasing amounts of G3P. **d** Purified LCK protein was analyzed by SDS–PAGE, followed by Coomassie blue staining. **e** BIAcore measurement of the interaction between G3P and the purified LCK. **f** The interaction between LCK and G3P were speculated using three-dimensional structures. **g** Schematic of LCK structure. **h** Purified region LCK (60-240AAs) was analyzed by SDS–PAGE, followed by Coomassie blue staining. **i** BIAcore measurement of the interaction between G3P and the purified region. **j** Purified region LCK(240-509AAs) was analyzed by SDS–PAGE, followed by Coomassie blue staining. **k** BIAcore measurement of the interaction between G3P and the purified region. **l** Purified region LCK(230-390AAs) was analyzed by SDS–PAGE, followed by Coomassie blue staining. **m** BIAcore measurement of the interaction between G3P and the purified region. **n** Purified region LCK(330-509AAs) was analyzed by SDS–PAGE, followed by Coomassie blue staining. **o** BIAcore measurement of the interaction between G3P and the purified region

The Tyr505 site at the C-terminus of LCK is dephosphorylated, thus allowing LCK to be activated by trans-autophosphorylation at Tyr394.^40^ Intriguingly, we observed that G3P dose-dependently inhibited LCK phosphorylation at Tyr394 while enhancing phosphorylation at Tyr505 (Fig. 6a, b and Supplementary Fig. 7a). To clarify the regulation of these sites by G3P, we purified LCK and its mutants (Y394F and Y505F) (Supplementary Fig. 7b). Preventing phosphorylation at Tyr394 (Y394F) greatly decreased LCK activity, while blocking Tyr505 phosphorylation (Y505F) enhanced LCK activity, but the addition of G3P couldn’t remarkably inhibit LCK (Y394F) and LCK (Y505F) (Supplementary Fig. 7c). Immunoblotting showed that the Y394F mutation had no impact on G3P-induced enhancement of Tyr505 phosphorylation, while the mutation (Y505F) abolished the inhibitory effect of G3P on Tyr394 phosphorylation (Fig. 6c). This indicates that G3P inhibited Tyr394 autophosphorylation by promoting Tyr505 phosphorylation.

It is reported that asparagine, disulfiram and some inhibitors can directly bind to LCK.^41,42^ Next, we demonstrated the interaction of LCK and G3P using surface plasmon resonance (BIAcore) experiment (Fig. 6d, e), the same results were obtained by isothermal titration calorimetry (ITC) (Supplementary Fig. 7d, e). Next, we constructed a computational 3D model of the complex structure using the X-ray crystal data, and docking simulation results revealed the interaction between LCK and G3P (Fig. 6f). To pinpoint the specific location of LCK binding to G3P, we constructed LCK deficient mutants (Fig. 6g), then surface plasmon resonance (BiAcore) experiment revealed that G3P bound to the region spanning AA330 to 509 within the LCK’s kinase domain (Fig. 6h–o and Supplementary Fig. 7f), which was further confirmed by isothermal titration calorimetry (ITC) (Supplementary Fig. 7g–n). Hence, G3P inhibits LCK activity by binding to the AA330-509 region.

### Targeting VCP combined with anti-PD1 modulates the TME of HCC

**Fig. 7:**
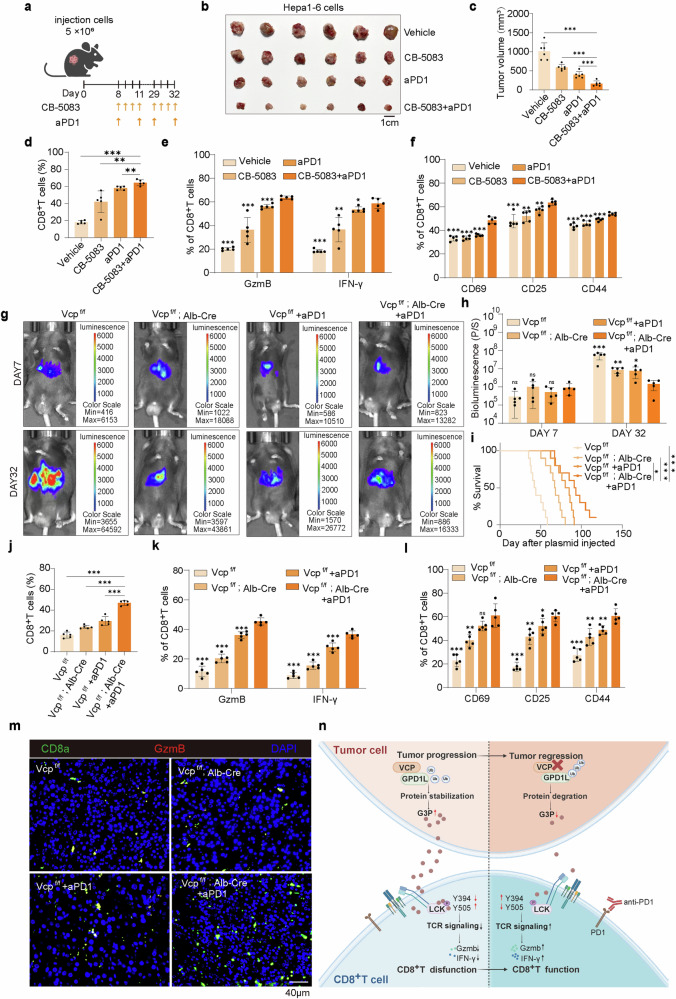
Targeting VCP combined with anti-PD1 modulates the TME of HCC. **a** Treatment schema of Hepa1-6 tumor-bearing C57BL/6 mice. Schematic created with BioRender.com. **b** Hepa1-6 tumors were dissected from the C57BL/6 mice treated with vehicle, CB-5083, aPD1, or both (*n* = 6/group). **c** Data from (**b**). **d**–**f** The percentage of CD8^+^T cells, cytokines production (Granzyme B, IFN-γ), and activation indicators (CD69, CD25, CD44) in Hepa1-6 tumors were measured by flow cytometry analysis (*n* = 5/group). **g**, **h** Bioluminescence images of conditional knockout mice at indicated time points after plasmid injection. Bioluminescence signal at indicated time points post plasmid injection in Vcp conditional knockout mice. **i** Kaplan–Meier survival curve of conditional knockout mice treated as in (**g**). Log-rank test. **j**–**l** The percentage of CD8^+^T cells, cytokines production (Granzyme B, IFN-γ), and activation indicators (CD69, CD25, CD44) with spontaneous tumors in Vcp conditional knockout mice were measured by flow cytometry (*n* = 5/group). **m** Spontaneous tumor sections of conditional knockout mice were stained with multiple immunofluorescence. **n** Schematic summary graph of the main conclusion of the study. Tumor cells VCP promotes the generation of G3P through stabilizing GPD1L, which then impairs TCR signaling and causes CD8^+^T cells dysfunction. Schematic created with BioRender.com. Data are presented as mean values ± SD. Statistical significance was determined using two-sided t-tests, *P < 0.05, **P < 0.01, and ***P < 0.001

Given that immune cells share many metabolic ways that are critical for tumor cells survival, the therapeutic window of metabolic drugs is usually narrow, which may lead to severe toxicity.^43^ Metabolic plasticity remains a challenge when targeting tumor-specific metabolism.^44^ Due to the lack of inhibitors targeting metabolite G3P as well as metabolic enzyme GPD1L, we targeted upstream VCP as a therapeutic target. PD-1 triggers T cell inactivation by phosphorylating tyrosine residues in its intracellular domain, which leads to the recruitment of the tyrosine phosphatase SHP-2. This process subsequently reduces the phosphorylation of key components in the TCR signaling pathway, resulting in diminished downstream activation signals and a decrease in cytokine production.^45^ VCP inhibition restores TCR signaling and promotes CD8^+^T cells’ proliferation and effector function, which provides the possibility for restoring T cells activity after PD-1 treatment. Therefore, We proposed that targeting VCP inhibition could work in synergy with anti-PD1 therapy to reshape the TME (Fig. 7a).

Indeed, treatment of mice harboring Hepa1-6 tumors with the specific VCP inhibitor CB-5083 combined with anti-PD1 suppressed tumor growth without mouse weight loss compared to monotherapy or control groups (Fig. 7b, c and Supplementary Fig. 8a, b). Flow cytometry proved that the combination heightened the infiltration, effector function, and CD8^+^T cells activation (Fig. 7d–f and Supplementary Fig. 8c, d). Multiple immunofluorescence and immunohistochemistry confirmed higher CD8^+^T cell infiltration and GzmB staining intensity in combination treatment group (Supplementary Fig. 8e, f). In addition, G3P levels in tumor interstitial fluid notably decreased after CB-5083 combined with anti-PD1 treatment (Supplementary Fig. 8g).

Significantly, to produce a murine strain with liver-specific deletion of Vcp (Vcp^f/f^; Alb-Cre), we crossed Vcp^f/f^ mice with Alb-Cre mice, followed by Vcp^f/f^; Alb-Cre and Vcp^f/f^ mice were used to establish spontaneous HCC model by tail vein plasmid injection (Supplementary Fig. 8h, i). Compared with Vcp^f/f^, anti-PD1 treated Vcp^f/f^; Alb-Cre mice showed obviously decreased tumor development and extended survival (Fig. 7g–i and Supplementary Fig. 8j). Flow cytometry analysis revealed increased numbers, cytokine expression, and activation of CD8^+^T cells in Vcp^f/f^; Alb-Cre mice treated with anti-PD1 (Fig. 7j–l). Multiple immunofluorescence staining showed higher CD8^+^T cell granules and GzmB staining intensity in tumors of Vcp^f/f^; Alb-Cre mice treated with anti-PD1 (Fig. 7m), corroborated by immunohistochemistry (Supplementary Fig. 8k). Similarly, G3P levels in tumors of Vcp^f/f^; Alb-Cre mice treated with anti-PD1 were lower than those in other groups (Supplementary Fig. 8l). The findings suggest that inhibiting VCP can augment the effectiveness of anti-PD1 immunotherapy and optimize the immune microenvironment in HCC.

## Discussion

Tumors evade immune surveillance by forming a complex immunosuppressive environment. Elucidating the underlying mechanisms and subsequent remodeling of the immunosuppressive microenvironment to direct appropriate combination therapy continues to pose a significant obstacle for HCC patients. In our research, it was discovered that the increased level of VCP has a close association with the suppressive immune microenvironment and unfavorable prognosis in HCC patients. To be specific, we demonstrated that VCP stabilizes downstream GPD1L and promotes the accumulation of G3P, which then binds to the LCK protein of the TCR signaling pathway, inducing the phosphorylation of LCK Tyr505 and inhibiting its activity, thereby impairing CD8^+^T cells activation and rendering them ineffective in exerting their cytotoxic effects. This mechanism establishes a direct link between tumor metabolism and immune suppression in HCC. Meaningfully, treatment with the VCP inhibitor CB5083 or Vcp knockout in vivo, combined with anti-PD1 therapy, resulted in significant tumor suppression in mouse HCC models (Fig. 7n). The findings show that targeting VCP could represent a new therapeutic avenue for HCC by addressing both tumor metabolism and immune dysfunction, offering a synergistic strategy that could improve the efficacy of existing immunotherapies and provide broader applications in cancer treatment.

Regarding the mechanism of VCP in HCC, VCP can promote HCC progression through the PI3K/AKT/mTOR pathway.^46^ However, our research shifts the focus to the role of VCP in the TME and its potential in combination therapy, which offers a novel direction. Several studies have reported the close link between the HCC tumor microenvironment and immunotherapy, with mechanisms involving various factors. For instance, SOX18-mediated transcriptional dysregulation is a critical tumor-intrinsic mechanism in creating an immunosuppressive TME, and targeting SOX18 signaling pathways could strengthen the effect of anti-PD-L1 treatment.^47^ In addition, inhibiting GPR109A or supplementing glutamine has been shown to reduce immunosuppression and slow tumor growth.^48^ Furthermore, research integrating cutting-edge technologies, such as Stereo-seq and scRNA-seq, has unveiled the complex and dynamic immune landscape of human liver cancer, shedding light on how the tumor microenvironment evolves and interacts with immune cells.^49^ In contrast, our investigation did not delve into the impacts of VCP on other immune cells or their interactions, which could be a limitation. Considering their pivotal role in immune evasion, macrophages, regulatory T-lymphocytes, and myeloid-derived suppressive cells are of utmost importance, the prospective function of VCP in regulating these cells within the TME demands additional exploration. Despite these limitations, we focused on VCP’s role in the HCC tumor microenvironment, exploring how it suppresses CD8^+^T cells function via downstream metabolites, particularly G3P, which links tumor metabolism with immune dysfunction. By identifying this pathway, we offer a new perspective on therapeutic strategies, emphasizing the importance of targeting regulators like VCP to enhance immunoreaction and enhance the effect of combination therapies in HCC.

The mechanism through which metabolic changes in TME contribute to T cell dysfunction is still unclear. We determined the accumulation of G3P, a downstream metabolite of VCP, which allows G3P to enter CD8^+^T cells to inhibit the excitation of TCR signaling. Asparagine strengthens the kinase activity of LCK in the TCR signaling pathway by binding to LCK.^41^ Interestingly, we demonstrated that G3P also binds to LCK, but exerts the opposite effect by restraining T cell activation, ultimately leading to CD8^+^T cells disability. Mechanistically, VCP promotes G3P accumulation by stabilizing GPD1L, indicating that GPD1L may be a valuable biomarker for HCC. G3P supports lipid synthesis to promote multiple cancer progression.^30,31^ The accumulation of G3P not only drives tumor growth through its metabolic contributions but also profoundly impacts immune evasion by impairing T cell-mediated responses. We demonstrated a detrimental effect of G3P on CD8^+^T cells, as its interaction with LCK inhibits TCR signaling, rendering these cytotoxic cells ineffective in targeting tumor cells. This dual role of G3P, as both a facilitator of tumor progression and an immune suppressor, underscores its prospective utility as a treatment target. In addition, we started with the upstream VCP of G3P and ultimately used VCP as a target for combined immunotherapy. This strategy, involving the inhibition of VCP to regulate G3P levels, provides new theoretical support for tumor immunotherapy and key clues for developing more effective treatment methods. By bridging the gap between tumor metabolism and immune dysfunction, our findings lay the groundwork for innovative combination therapies, particularly in HCC. Future studies should further explore the broad applications of targeting the VCP-G3P axis in other cancer types.

While our study suggests therapeutic potential in combining VCP inhibition with anti-PD1 for HCC, clinical studies in patient cohorts, and other HCC models, are needed to further verify this hypothesis. To translate these discoveries into clinical application, a thorough assessment of the safety profile, effectiveness, and appropriate dosage of VCP inhibitors, exemplified by CB5083, in conjunction with anti-PD1 treatment, is necessary. In addition, the heterogeneity observed in HCC tumors and their associated immune microenvironment underscores the necessity for stratified approaches that identify patient subgroups most likely to benefit from this combination therapy. Significantly, our research reveals a possible application of the combined therapy of targeted VCP and anti-PD1 in other cancer varieties, as VCP is universally highly expressed in pan-cancer, and low anti-PD1 response rates are also present in various cancer types like melanoma, renal cancer and colorectal cancer. Nevertheless, these applications necessitate further investigation to determine the consistency of VCP’s mechanisms across different cancers. Future studies should explore the broader implications of VCP inhibition, including its effects on non-T cell populations and other components of the tumor microenvironment. In addition, clinical trials incorporating biomarkers, such as GPD1L expression or G3P levels, could guide patient selection, ensuring the effective deployment of this combination therapy.

In conclusion, our study recognized VCP as a significant biomarker for forecasting the results of HCC patients requiring immunotherapy. It also uncovered a novel mechanism that exhibits the synergistic effectiveness of the combined treatment of targeting VCP and anti-PD1 for HCC. This combination therapy remarkably promotes the CD8^+^T cells infiltration, remodels the TME, and ultimately leads to regression of HCC tumors. In terms of mechanism, VCP inhibition can promote the ubiquitination and degradation of the downstream metabolic enzyme GPD1L, thereby reducing the accumulation of metabolite G3P, enhancing TCR signaling pathway activation, and restoring CD8^+^T cells effector function. Our data present initial clinical proof and mechanistic understanding for a new combined approach to improve the clinical effectiveness of anti-PD1 antibody-centered immune therapy in HCC.

## Materials and methods

### Clinical patients

Samples in paraffin sections, utilized in this research, were sourced from patients at the First Affiliated Hospital of the University of Science and Technology of China. Neither the clinical diagnosis nor the treatment of the patients was impacted by the acquisition of these specimens, nor did it impose any physical or mental strain upon them. The hepatocellular carcinoma tissue microarray (TMA) of 90 HCC tumors and surrounding tissue was used. The tissues were obtained from patients undergoing surgery in Department of Hepatobiliary Surgery, First Affiliated Hospital of the University of Science and Technology of China. With the patients’ informed consent, all tissue samples were gathered. Approval for the research was granted by the Institutional Review Board of the center, with the approval number being 2024-KY544.

### Mouse experiments

C57BL/6, nude mice, C57BL/6JGpt-Vcp^em1Cflox^/Gpt, Alb-cre mice and OT-1 mice were procured from GemPharmatech and Hangzhou Ziyuan. Approval was granted for conducting the animal experiments, which adhered to the ethical principles outlined by the Animal Ethics Committee at the First Affiliated Hospital of USTC.

Regarding subcutaneous tumor model, after 3 days acclimatization, hepa1-6 cells, either shCtrl or shVcp-transfected, were implanted subcutaneously into nude or C57BL/6 mice at a cell count ranging from 3 to 5 million. About 4–5 weeks later, the tumors were extracted for investigation. When tumor volume reached 50 mm^3^, mice were randomly selected for subsequent antibody or drug treatment. For antibody injections, anti-CD8 mAb (Bioxcell, BE0117) (200 μg/mouse, twice a week) injection was started 1 week before tumor implantation. For combination therapy, anti-PD1 mAb (Bioxcell, BE0146) and IgG2a (Bioxcell, BE0089) (100 μg/mouse) were administered by intraperitoneal injection, CB-5083 (50 mg/kg) was administered by oral gavage.^50^

For the model of spontaneous HCC tumorigenesis, through the tail vein, a mixture of 11.4 μg pT3-EF1a-MYC-IRES-luciferase, 10 μg px330-sg-p53, 10 μg px330-Vcp, and 5.35 μg CMV-SB13 was administered.^32^ After injecting the mice with D-luciferin (GoldBio), IVIS Spectrum system was used to perform in vivo bioluminescence imaging to quantify liver tumor burden. The measurement of the signal intensity was carried out with Living Image.

To establish the Vcp^fl/fl^/Alb-Cre mouse model of spontaneous HCC, to generate Vcp cKO mice, Vcp^fl/fl^ mice were crossed with Alb-Cre mice. Male Vcp cKO mice aged 5–6 weeks were employed in the ensuing experiments.

### Cell lines and cell culture

Hepa1-6, HCCLM3, Huh7, HepG2, Hep3B, PLC/PRF/5, and HEK293T were cultivated in DMEM medium (Sigma), enriched with 10% fetal bovine serum (Sigma) and 1% penicillin-streptomycin antibiotic mix (Gibco). Hep3B, Huh7, and PLC/PRF/5 were procured from Accegen. HEK293T and hepa1-6 and were purchased from ATCC. HepG2 and HCCLM3 were gifted by the professor Hongyang Wang (SMMU). Primary CD8^+^T cells were cultured in RPMI-1640 (Sigma), supplemented with 10% fetal bovine serum (Sigma), 1% penicillin-streptomycin (Gibco), and interleukin-2 at a concentration of 10 ng/mL.^51^ Primary T cells were stimulated with antibodies against CD3 and CD28, with a concentration of 2 µg/mL for each.^13^ Jurkat cells were maintained in RPMI-1640 (Sigma), supplemented with 10% fetal bovine serum (Sigma) and 1% penicillin-streptomycin (Gibco). These cells were incubated in a humidified environment at 37 °C, with 5% CO_2_ present.

### Plasmids

The plasmids pcDNA3.1-HA, pcDNA3.1-FLAG, pGEX-6P-1, and pET-22b were provided by Professor Qingsong Hu. The plasmids pLKO.1-puro, pMD2.G, and psPAX2 were generously provided by Professor Mian Wu. The CMV-SB13 vector was received from Professor Amaia Lujambio. In addition, pT3-EF1A-MYC-IRES-Luc and pX330p53 were acquired from Addgene. pCMV-His-UB (P4836) and pLV3-CMV-OVAL(chicken)-CopGFP-Puro (P43339) were purchased from Miaoling Biology. For protein expression, VCP and GPD1L cDNAs were cloned into pGEX-6P-1 and pET-22b vectors, respectively. The shRNAs are listed in Supplementary Table S1.

### Cell transfection and virus infection

Lipofectamine 3000 (Thermo) was used for transient transfection. For viral infection purposes, HEK293T cells were transfected with a combination of plasmids: psPAX2, pMD2.G, and either PLKO.1 or pCMV-3xFLAG, resulting in the production of viruses. The viruses were subsequently filtered and collected for use in infections. The cells were then subjected to selection using appropriate concentrations of puromycin (APExBIO).

### CD8^+^T cell isolation

Isolation of naive CD8^+^T cells from mouse spleens was accomplished utilizing a specific isolation kit (Miltenyi Biotec, 130-096-543), specifically designed for this purpose. Isolation of human CD8^+^T cells involved a two-step process: initially, peripheral blood mononuclear cells were separated by gradient centrifugation, and subsequently, CD8 microbeads (Miltenyi Biotec, 130-093-244) were employed for further purification.

### RNA isolation and qPCR

Cellular RNA was isolated utilizing the GeneJET RNA Purification Kit (Thermo Fisher). This RNA was subsequently reverse-transcribed into cDNA with the assistance of PrimeScript RT Master Mix supplied by Takara. For the purpose of quantitative PCR (qPCR), TB Green Premix Ex Taq II, also sourced from Takara, was utilized in experimental procedure, with β-Actin acted as the internal control. The primers are listed in Supplementary Table S1.

### Antibodies

Anti-CD8a (98941), anti-CD8a (85336), anti-GranzymeB (46890), anti-SQSTM1 (5114), anti-His (2365), anti-GST (2622), anti-PI3Kinase (4249), anti-p-PI3Kinase (4228), anti-LCK (2752), anti-ZAP-70 (3165), anti-p-LCK (Tyr505) (2751), anti-p-ZAP-70 (2717), anti-LAT (45533), anti-p-LAT (3584), anti-β-Actin (3700), anti-Flag (14793), and anti-HA (3724) are from Cell Signaling Technology. Anti-VCP (MA3-004), Alexa Fluor 488/555 (A11001, A21428) are from Thermo Fisher Scientific. Anti-p-LCK (Y394) (MAB7500) is from R&D. Anti-GPD1L (17263-1-AP), anti-GPD1 (13451-1-AP), and anti-OVA (67614-1-Ig) are from Proteintech. Anti-mouse CD45 (103137), anti-human/mouse GranzymeB (515406), anti-mouse IFN-γ (163504), anti-mouse CD69 (104505), anti-mouse CD25 (102024), anti-human/mouse CD44 (103008), anti-mouse CD8a (100734C), anti-mouse CD3 (100236), anti-human CD45 (304012), anti-human CD3 (317332), anti-human CD8a (344710), anti-human IFN-γ (383304), anti-human CD69 (310922), and anti-human CD25 (302604) are from Biolegend.

### Drug treatment

CB-5083 (HY-12861) and NMS-873 (HY-15713) were obtained from MCE. Cycloheximide (S7418), MG132 (S2619), PP2 (S7008), and Chloroquine (S6999) were purchased from Selleck.

### Immunoprecipitation

The cells were instantly immersed in liquid nitrogen for rapid icy and then lysed in a buffered solution comprising 20 mM HEPES adjusted to pH 7.4, 150 mM NaCl, 1 mM EDTA, and 0.1% Triton X-100. In addition, cocktail (Sigma, PPC1010) was included in the lysis buffer. The cellular lysates underwent clarification through a centrifugation process conducted at a temperature of 4 °C for a duration of 30 min, then incubated at the same temperature for 4 h with magnetic beads (Thermo, 88802, 88837, and A36797) or specific primary antibodies. The beads underwent a washing procedure using a buffer solution that consisted of 50 mM Tris-HCl adjusted to a pH of 7.4, 150 mM NaCl, and 1 mM EDTA, along with the inclusion of the protease inhibitor. Eventually, to elute the proteins that were bound to the beads, a boiling step was carried out in a loading buffer solution, and then immunoblotting was employed for analysis.

### Western blot

Cells were first quantified utilizing a cell counter, subsequently lysed in RIPA buffer (Beyotime) that also contained a protease inhibitor, and then incubated on ice for a period of 30 min. After centrifugation, supernatants were retrieved, mixed with loading buffer, and heated. The proteins were then resolved using gradient gel (Beyotime) and subsequently imprinted onto PVDF membrane (Qiagen). The stripes were subjected to blocking with a solution of 5% defatted milk for a duration ranging from 30 to 60 min, followed by overnight incubation with primary antibodies and a 40-min soaking with secondary antibodies. Target bands were visualized using a ChemiDoc imaging system (BioRad) after being detected with ECL Western Blotting Substrate (Thermo).

### Immunohistochemistry

Tissue sections were baked in an oven for 30 min, deparaffinized in xylene, hydrated through graded alcohols, and underwent a process of antigen retrieval employing Antigen Unmasking Solution sourced from Vector Labs. Prior to the overnight incubation with primary antibodies at a temperature of 4 °C, sections were blocked using a solution of 10% goat serum for a duration of 30 min. Post-washing with PBST, the sections underwent a 1-h incubation process utilizing a secondary antibody, which was biotin-labeled and sourced from Vector Labs. This was followed by a 30-min treatment with avidin-biotin complex reagent, also provided by Vector Labs. For visualization, a DAB kit was employed. Subsequently, the sections underwent hematoxylin staining, dehydration through a series of alcohol and xylene solutions, and were finally mounted using neutral balsam. The Histochemistry score (H-Score) was used for quantification.^52^ A higher value signified a greater comprehensive positive intensity in terms of both positive depth and positive quantity.

### Hematoxylin & eosin and immunofluorescence staining

Regarding H&E, the tumor tissues were initially fixed in formalin for a whole night and followed by embedding in paraffin. Microtome-cut sections were then stained with a combination of hematoxylin and eosin. In the case of immunofluorescence (IF) staining, cells were initially plated onto glass slides and fixed with 4% paraformaldehyde for 10 min. Permeabilization was accomplished using 0.2% Triton X-100 for 3 min. Slides blocking was carried out for 1 h in PBST containing 0.05% Tween-20 and 1% BSA sourced from Sigma. Primary antibodies were then incubated with the slides overnight at 4 °C. Following three washes with PBST, slides were incubated with secondary antibodies for 1 h, and ultimately mounted with DAPI from Vector Labs for visualization.

### Multiplex IF assay

Tumor sections underwent deparaffinization using xylene and were then rehydrated through a series of ethanol solutions. They underwent treatment with Antigen Unmasking Solution sourced from Vector Labs and incubated in a 3% hydrogen peroxide solution. Within a humidified chamber, the primary antibodies were incubated for a duration of 60 min. Afterwards, a 30-min period of incubation was conducted using the corresponding secondary antibody, which was conjugated with horseradish peroxidase and sourced from CST. Thereafter, tissue sections were incubated with fluorophore-conjugated TSA Plus amplification reagent (CST) for 10 min. Before next antibody incubation the sections were again treated with Antigen Unmasking.^53^ Finally, an antifade reagent containing DAPI (Vector Labs) was applied for sealing. The Zeiss Axio Imager M2m was utilized to capture the images.

### Confocal immunofluorescence imaging

G3P (Sigma, 94124) was added to CD8^+^T cells and Jurkat cells for a 30-min incubation period, with control groups receiving no G3P. Subsequently, all cells were stimulated with anti-CD3 (at a concentration of 2 µg/mL) and anti-CD28 (also at 2 µg/mL). Immunofluorescence (IF) staining procedures were then performed as previously outlined. The Zeiss LSM 800 confocal laser scanning microscope was utilized to capture pictures, and quantification was performed using ImageJ software.^54^

### Protein purification

GST/GST-fusion and His-fusion plasmids were transformed into competent BL21 cells. Protein purification was carried out using the GST and His-tag protein purification kit (Beyotime). Cells were lysed via ultrasonic fragmentation after overnight culture. The supernatant was harvested through centrifugation and subsequently incubated with BeyoGold™ resin specific for GST-tag or His-tag purification at a temperature of 4 °C for 1 h. Following this, the resin was washed with a buffer solution designed for this purpose. Finally, the fused proteins were eluted using elution buffer for subsequent analysis.

### GST pull-down

A lysis buffer, comprising 50 mM Tris-HCl at pH 7.4, 150 mM NaCl, 1 mM EDTA, 5% glycerol, 0.1% Triton X-100, and 1 mM DTT, was supplemented with cocktail. GST fusion proteins (10 μg in 15 µL) and His fusion proteins (ranging from 10 to 20 μg) were mixed in this buffer and incubated at 4 °C for 4 h. Following three washes with a wash buffer (containing 150 mM NaCl, 1 mM EDTA, 0.2% Triton X-100, 50 mM Tris-HCl, and 1 mM DTT), also containing cocktail, the proteins were centrifuged, boiled, and subjected to immunoblot analysis.

### Detection of cell viability

The CCK8 assay kit from DOJINDO and flow cytometry (FC) were utilized to assess proliferation of CD8^+^T cells, adhering strictly to the manufacturer’s instructions. For the purpose of conducting a cell growth curve assay, the cells were dispensed into 96-well microplates for plating. Viability was assessed daily over three days. Cells were pre-stained with CFSE (BD, 565082) prior to culture and analyzed by FC after three days.^55^

### Elisa assay

Supernatants from CD8^+^T cell cultures were analyzed to determine IFN-γ concentrations using an ELISA kit (Beyotime, PI507). G3P levels were measured using an ELISA kit (Abcam, ab174094). Phosphorylation of Tyr394 on LCK was quantified with a Phospho-Lck (Y394) ELISA kit (Abcam, ab279848).

### scRNA-Seq analysis

Hepatocellular carcinoma tissues from mouse spontaneous tumor models were dissected into 1 mm^3^ pieces. Following grinding, enzymatic digestion, and filtration, single-cell suspensions were prepared. The sequencing library was first built, and subsequently, high-throughput sequencing was performed on this library utilizing Illumina NovaSeq 6000 sequencing platform. Next, the obtained fastq files were employed for additional investigation. The sequencing data were processed, including quality control, single-cell data screening, gene expression calculation, cell clustering and finally the cell types, gene expression differences, cell response differences and other information were obtained.

### Flow cytometry

Using mechanical techniques, tumors and CD8^+^T cells were dissociated to form single-cell suspensions. Regarding surface staining, cells were incubated with relevant antibody in the dark for a period of 1 h. Before FC analysis, washing was carried out twice with staining buffer. About the intracellular staining, cells were initially fixed with 4% paraformaldehyde for a duration of 20 min, and subsequently permeabilized using 1% Triton X-100 for an additional 10 min.

### Cytotoxicity and apoptosis assays

Plates were used to stimulate CD8^+^T cells for a period of 48 h with anti-CD3 (at a concentration of 2 µg/mL), anti-CD28 (at 2 µg/mL), and interleukin-2 (IL-2) at a concentration of 10 ng/mL. Following activation, then the CD8^+^T cells were cultured with cancer cells in 96-well plates for a duration of 24 h. Cytotoxic efficiency was assessed using an LDH Cytotoxicity Assay Kit (Beyotime, C0017) and an Annexin V-FITC/PI Apoptosis Kit (Biosharp, BL110A).^56^

### Tumor intestinal fluid (TIF) isolation

Tumors from mice or patients were processed within 30 min post-extraction. Samples underwent three washes with PBS, succeeded by mincing and centrifugation at 400 × *g* for 20 min. TIF was then collected by passing through a 20 μM nylon net filter.^57^

### Metabonomics analysis

The cells culture medium of shCtrl or shVCP HCCLM3 cells was collected, rapidly frozen in liquid nitrogen and analyzed by LC-MS/MS. Metabolomics analysis was accomplished in the laboratory or with the assistance of Biotree. In brief, 400 μl of extract solution was added to each sample. The mixture was vortexed for 30 s, followed by sonication for 10 min and incubation at −40 °C. Afterward, the sample was centrifuged at a speed of 10,800 times the force of gravity for 15 min, yielding the supernatant. For the purpose of LC-MS/MS analysis, a UHPLC system manufactured by Thermo Fisher Scientific, featuring a UPLC BEH Amide column, was interfaced with a Q Exactive HFX mass spectrometer.

### BIAcore analysis

Interactions between proteins and small molecules were evaluated utilizing a surface plasmon resonance (SPR) assay on a Biacore 8K platform. In brief, the purified proteins were fixed onto CM5 sensor chip (Cytiva) in sodium acetate buffer adjusted to a pH of 4.5. Meanwhile, G3P was dissolved in varying volumes of PBS buffer to establish a range of concentration gradients.^58^ The experimental results were analyzed using Biacore Insight Evaluation Software.

### Isothermal titration calorimetry

The purified proteins and G3P were diluted in PBS buffer. After the sample cell and titration needle were cleaned with ddH_2_O, samples were loaded. The titration was set at a temperature of 25 °C, a reference power of 5 μcal/s, and a volume of 2 μl. After titration, the machine was cleaned and shut down. Experiment was performed using MicroCal iTC200. The results were analyzed using MicroCal PEAQ-ITC Analysis Software.

### Molecular docking

The protein crystal structure, obtained from the PDB database with the identifier 2of2, was utilized for molecular docking. Three-dimensional structure of HMDB0000126 was sourced from PUBCHEM. Prior to docking, energy minimization of the structure was conducted using the MMFF94 force field. AutoDock Vina 1.2.3 software was used in this study.^59^ Prior to the docking operation, the receptor protein was readied by means of PyMol 2.5.5.^60^ This process involved the elimination of water molecules, salt ions, and small molecules from the protein structure. Following this, a docking box was established to encompass the entire protein. In addition, ADFRsuite version 1.0 was utilized to convert all prepared small molecules and receptor proteins into the PDBQT format, which is required by AutoDock Vina version 1.1.2.^61^ The binding mode was determined by selecting the docking conformation that scored the highest, and the obtained results were subsequently visualized utilizing PyMol, specifically version 2.5.5.

### Statistical analysis

Appropriate statistical tests, including paired t-tests, unpaired t-tests, log-rank tests, and two-way ANOVA, were utilized to ascertain statistical significance. These analyses were performed using Prism 8 software (GraphPad), with significance levels indicated as follows: ns (non-significant), *p < 0.05, **p < 0.01, and ***p < 0.001.

## Supplementary information

Supplementary Materials

Original WB

## Data Availability

The scRNA-seq data presented in the article have been uploaded to the GSA database with the identifier CRA016407. Furthermore, the metabolomics data generated and analyzed has been archived in MetaboLights database, accessible under the accession code MTBLS11917.
