# Supplementary material for: VCP downstream metabolite glycerol-3-phosphate (G3P) inhibits CD8+T cells function in the HCC microenvironment
Source: Signal Transduct Target Ther. 2025 Jan 24;10:26. doi: 10.1038/s41392-024-02120-8 (PMC11758394; doi:10.1038/s41392-024-02120-8)
Supplement: Supplementary file 1 — Supplementary Materials [file 41392_2024_2120_MOESM1_ESM.doc]

Supplementary Materials for

VCP downstream metabolite glycerol 3-phosphate (G3P)

inhibits CD8^+^T cells function in the HCC microenvironment

Cheng Cheng, Qingrui Zha, Linmao Sun, Tianming Cui, Xinyu Guo,

Changjian Xing, Zhengxiang Chen, Changyong Ji, Shuhang Liang, Shengwei

Tao, Junhui Chu, Chenghui Wu, Qi Chu, Xuetian Gu, Ning Zhang, Yumin

Fu, Shumin Deng, Yitong Zhu, Jiabei Wang, Yao Liu, Lianxin Liu

Correspondence to: Jiabei Wang (jbwang16@ustc.edu.cn) or Yao Liu (liuyao66@ustc.edu.cn)

or Lianxin Liu (liulx@ustc.edu.cn)

**This PDF file includes:**

Figures. S1 to S8

Tables S1 to S2


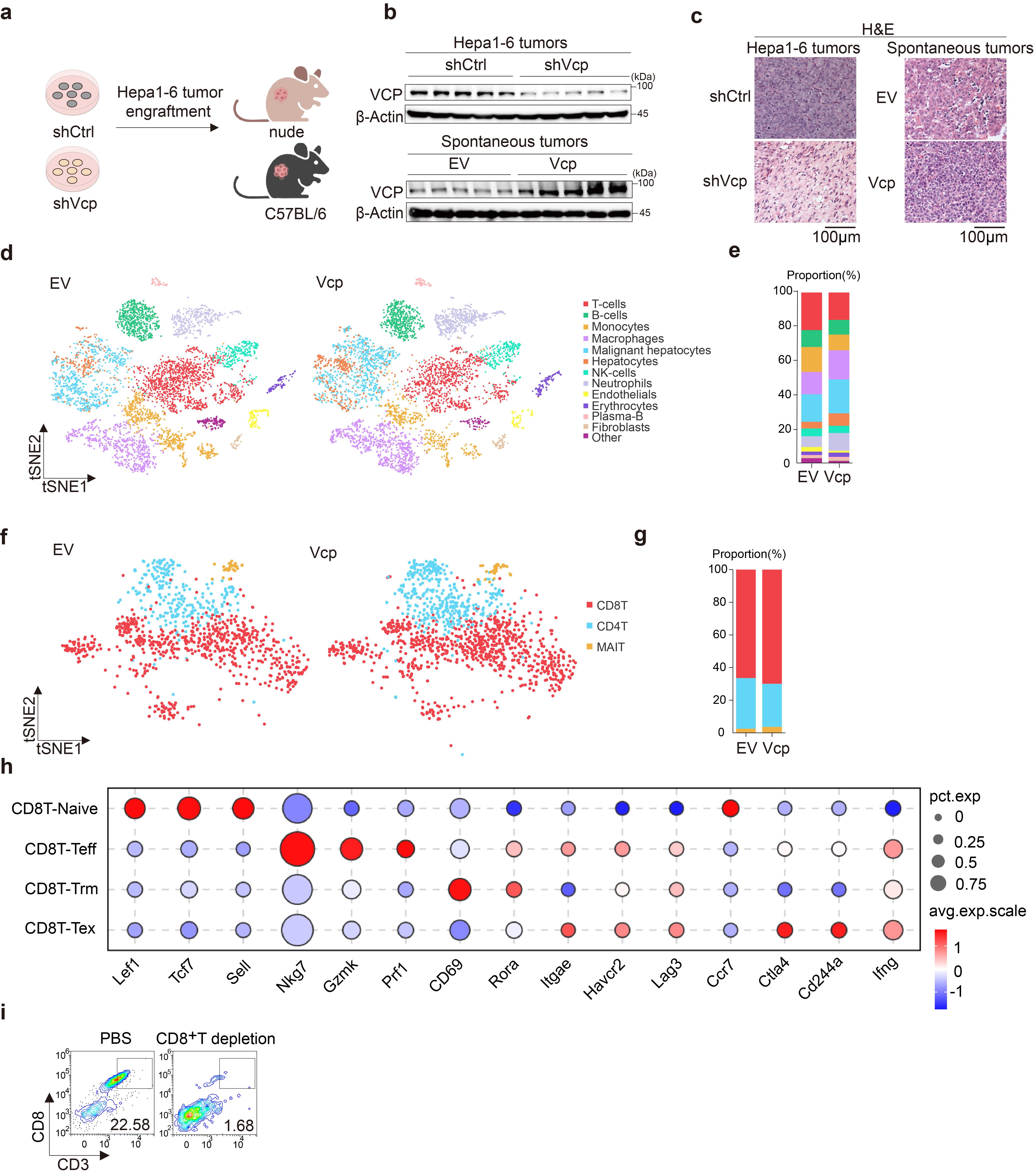


Figure. S1.

VCP affects effector CD8^+^T cells. (**a**) Related to Fig.1a. Experiment scheme of Hepa1-6-bearing nude and C57BL/6 mice. Schematic created with BioRender.com. (**b**)The expression of VCP in Hepa1-6 tumor and spontaneous tumor was analyzed by western blot (WB). (**c**) Hepa1-6 tumor and spontaneous tumor sections were subjected to Hematoxylin and Eosin (H&E) staining. (**d,e**) t-SNE analysis of scRNA-seq data of cells from HCC tumor tissues. (**f,g**) t-SNE analysis of scRNA-seq data of 3 subclusters from T cells. (**h**) Bubble plot of CD8^+^T cells subsets marker genes. (**i**) Flow cytometry analysis of CD8^+^T cell subset in splenocytes from anti-CD8a antibody-treated mice.


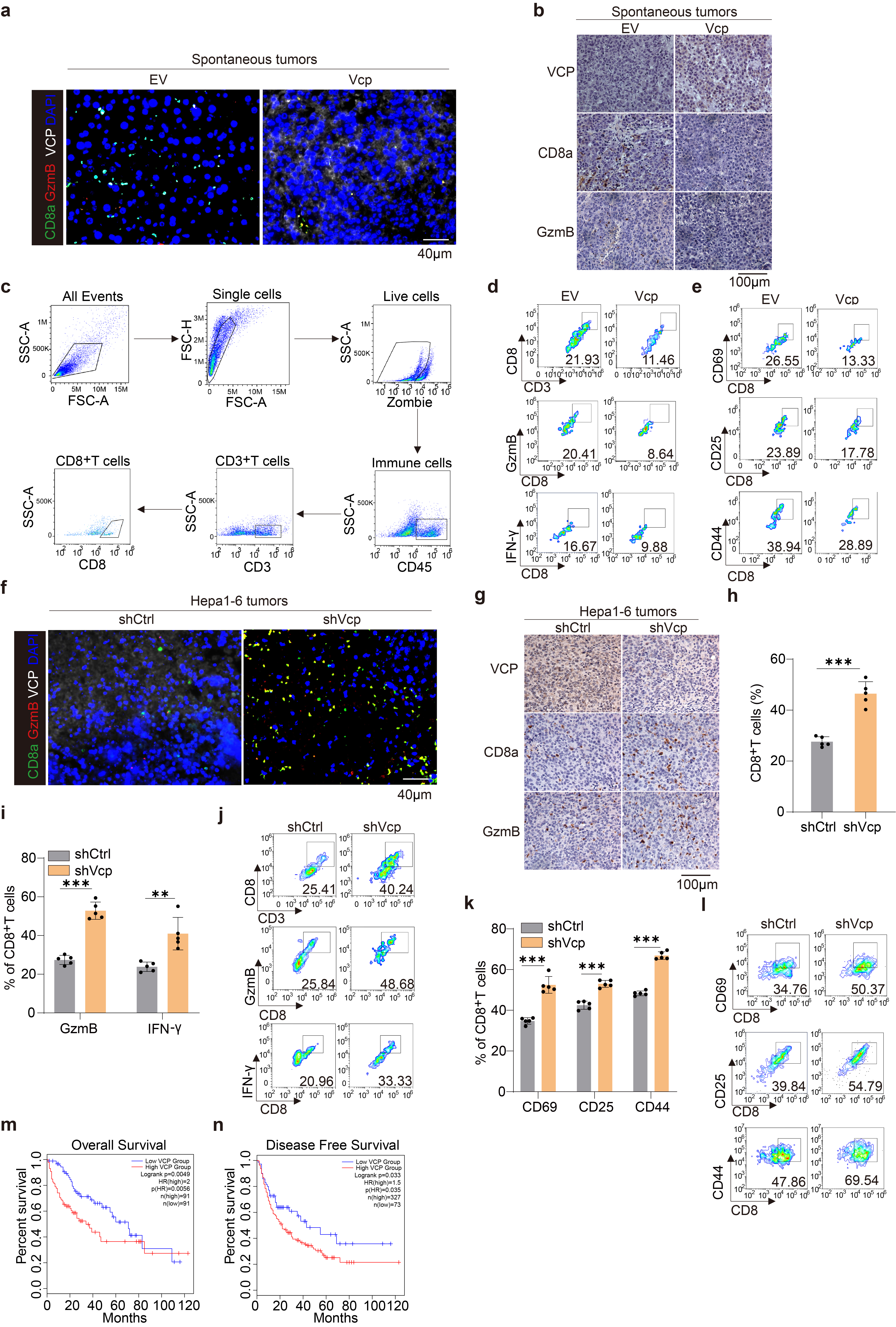


**Figure. S2.**

**VCP promotes HCC development by inhibiting CD8^+^T cells.**  (**a**) Spontaneous tumor sections were stained with multiple immunofluorescence for DAPI, CD8a, GzmB, and VCP. (**b**) Spontaneous tumor sections were stained with immunohistochemistry. (**c**) Gating strategies for flow cytometry of CD8^+^T cells. (**d,e**) Related to Fig. 1l-n. Representative contour plots of percentage of CD8^+^T cells, cytokines production, and activation indicators were measured by flow cytometry (n = 5/group). (**f,g**) Hepa1-6 tumor sections were stained with multiple immunofluorescence for DAPI, CD8a, GzmB, and VCP (**f**) and immunohistochemistry (**g**). (**h-l**) The percentage of CD8^+^T cells, cytokines production, and activation indicators in Hepa1-6 tumors were measured by flow cytometry (n = 5/group). (**m,n**) The clinical significance of VCP expression in overall and disease-free survivals in HCC patients was evaluated in the TCGA cohort. Data are presented as mean values ± SD. Statistical significance was determined using two sided t-tests, **P* < 0.05, ***P* < 0.01, and ****P* < 0.001.


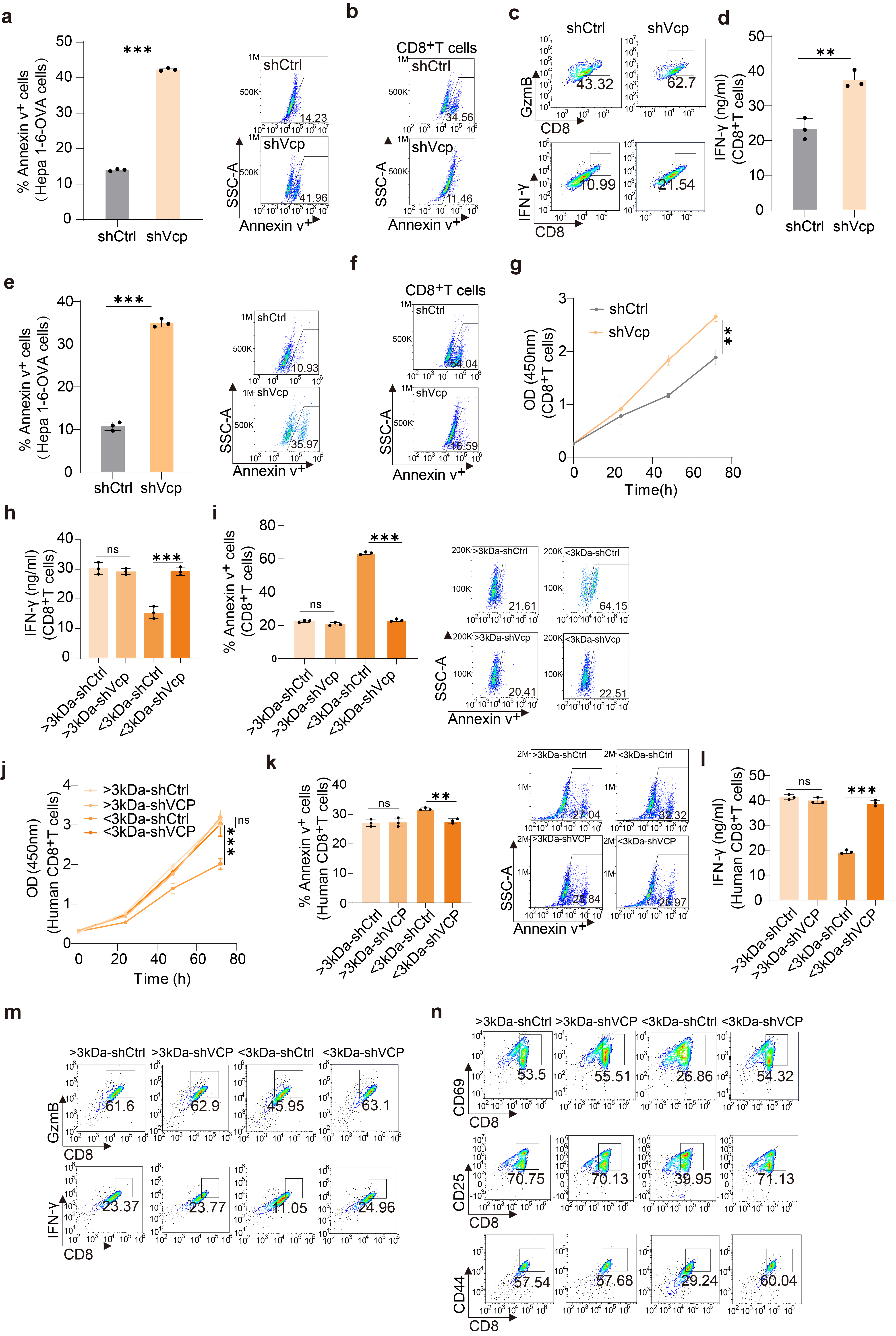


Figure. S3.

VCP of tumor cells indirectly inactivates CD8^+^T cells. (**a**) The percentage of Annexin v^+^ Hepa1-6-OVA cells co-cultured with CD8^+^T cells was determined by flow cytometry (n = 3). (**b**) Related to Fig. 2c. Representative scatter plots of Annexin v^+^ CD8^+^T cells co-cultured with tumor cells were measured by flow cytometry (n = 3). (**c**) Related to Fig. 2d. Representative contour plots of cytokines produced by CD8^+^T cells co-cultured with tumor cells were measured by flow cytometry (n = 3). (**d**) Secreted IFN-γ levels by CD8^+^T cells co-cultured with tumor cells (n = 3). (**e**)The percentage of Annexin v^+^ Hepa1-6-OVA cells co-cultured with CD8^+^T cells in Transwell system was determined by flow cytometry (n = 3). (**f**) Related to Fig. 2f. Representative scatter plots of Annexin v^+^ CD8^+^T cells in Transwell system were measured by flow cytometry (n = 3). (**g**) The proliferation of CD8^+^T cells in Transwell system was measured by CCK8 assay (n = 3). 2way ANOVA. (**h**) Secreted IFN-γ levels by CD8^+^T cells cultured with >/<3kDa CM produced by shCtrl or shVcp Hepa1-6-OVA cells (n = 3). (**i**) The percentage of Annexin v^+^ CD8^+^T cells cultured with >/<3kDa CM produced by shCtrl or shVcp Hepa1-6-OVA cells was determined by flow cytometry (n = 3). (**j**) The proliferation of human CD8^+^T cells cultured with >/<3kDa CM produced by shCtrl or shVCP HCCLM3 cells was measured by CCK8 assay (n = 3). 2way ANOVA. (**k**) The percentage of Annexin v^+^ human CD8^+^T cells cultured with >/<3kDa CM produced by shCtrl or shVCP HCCLM3 cells was determined by flow cytometry (n = 3). (**l**) Secreted IFN-γ levels by human CD8^+^T cells cultured with >/<3kDa CM produced by shCtrl or shVCP HCCLM3 cells (n = 3). (**m**) Related to Fig. 2q. Representative contour plots of cytokines produced by human CD8^+^T cells cultured with >/<3kDa CM produced by shCtrl or shVCP HCCLM3 cells were measured by flow cytometry (n = 3). (**n**) Related to Fig. 2r. Representative contour plots of activation indicators in human CD8^+^T cells cultured with >/<3kDa CM produced by shCtrl or shVCP HCCLM3 cells were determined by flow cytometry (n = 3). Data are presented as mean values ± SD. Statistical significance was determined using two sided t-tests, **P* < 0.05, ***P*< 0.01, and ****P* < 0.001.


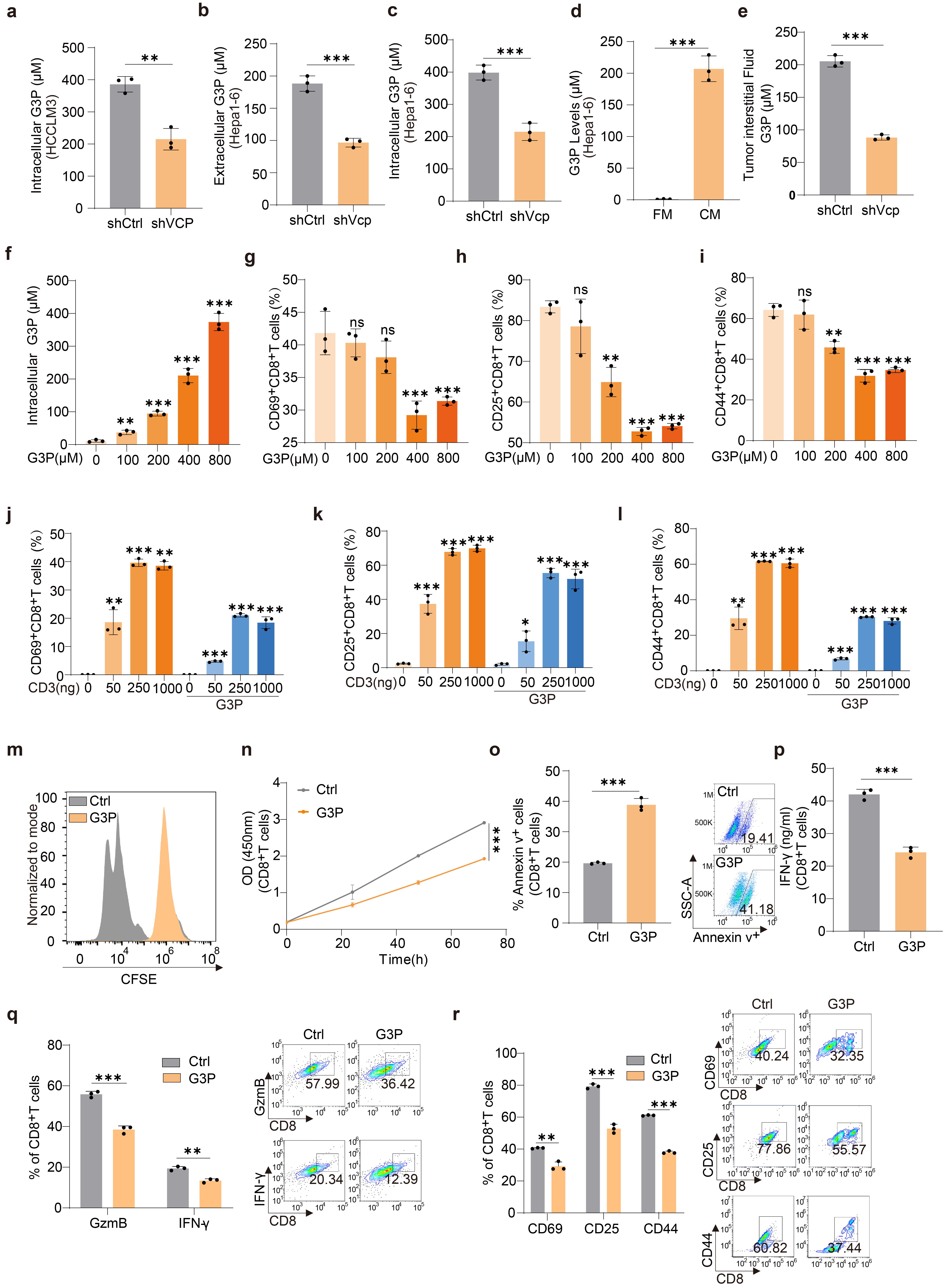


Figure. S4.

The downstream metabolite of VCP is G3P. (**a**) Intracellular G3P levels in shCtrl or shVCP HCCLM3 cells (n = 3). (**b**) Extracellular G3P levels in shCtrl or shVcp Hepa1-6 cells (n = 3). (**c**) Intracellular G3P levels in shCtrl or shVcp Hepa1-6 cells (n = 3). (**d**) G3P levels in fresh medium (FM) and conditional medium (CM) from Hepa1-6 cells (n = 3). (**e**) G3P levels in interstitial fluid of subcutaneous tumors (n = 3). (**f**) Intracellular G3P levels in CD8^+^T cells treated with gradient concentrations of G3P. Analysis was performed after 24 h (n = 3). (**g-i**) The expression of activation indicators in CD8^+^T cells treated with gradient concentrations of G3P was determined by flow cytometry. Analysis was performed after 24 h (n = 3). (**j-l**) The expression of activation indicators in CD8^+^T cells stimulated with CD28 antibody (2μg/ml) and increasing amounts of anti-CD3 antibody in the presence or absence of G3P (n = 3). (**m**) The proliferation of CD8^+^T cells treated with G3P was measured by CFSE assay. (**n**) The proliferation of CD8^+^T cells treated with G3P was measured by CCK8 assay (n = 3). 2way ANOVA. (**o**) The percentage of Annexin v^+^ CD8^+^T cells treated with G3P was determined by flow cytometry (n = 3). (**p**) Secreted IFN-γ levels by CD8^+^T cells treated with G3P. Analysis was performed after 24 h (n = 3). (**q**) The percentage of cytokines produced by CD8^+^T cells treated with G3P was measured by flow cytometry (n = 3). (**r**) The expression of activation indicators in CD8^+^T cells treated with G3P was measured by flow cytometry (n = 3). Data are presented as mean values ± SD. Statistical significance was determined using two sided t-tests, **P* < 0.05, ***P*< 0.01, and ****P* < 0.001.


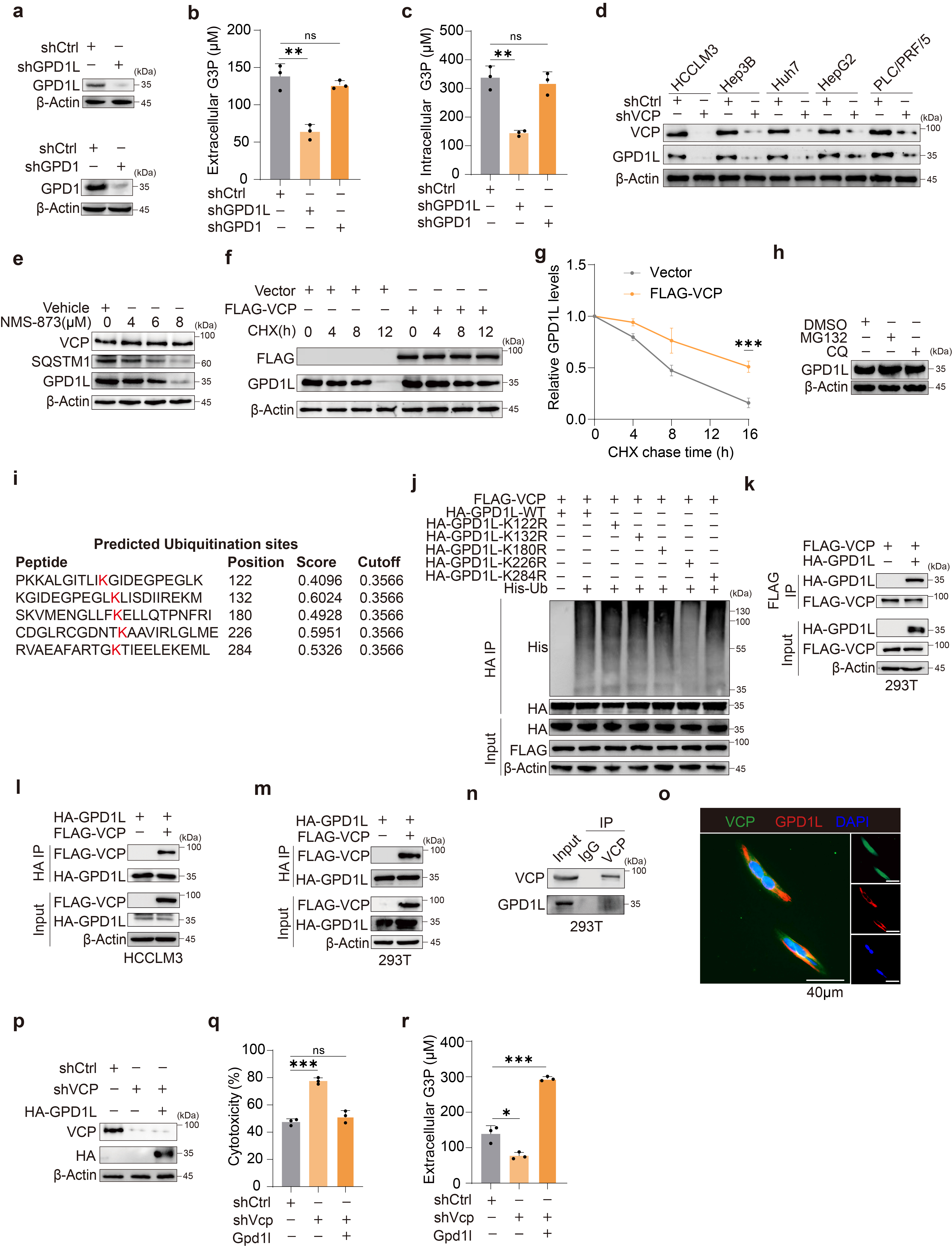


Figure. S5.

VCP interacts with GPD1L and stabilizes its expression. (**a**) Immunoblot analysis of the indicated proteins in HCCLM3 cells stably expressing shCtrl/shGPD1L/shGPD1. (**b**) Extracellular G3P levels in shCtrl/shGPD1/shGPD1L HCCLM3 cells (n = 3). (**c**) Intracellular G3P levels in shCtrl/shGPD1L/shGPD1 HCCLM3 cells (n = 3). (**d**) Immunoblot analysis of the indicated proteins in human HCC cell lines stably expressing shCtrl or shVCP. (**e**) Immunoblot analysis of the indicated proteins in HCCLM3 cells treated with gradient concentrations of NMS873 or Vehicle for 18h. (**f,g**) Immunoblot analysis of the indicated proteins in HCCLM3 cells transfected with Vector or FLAG-VCP and treated with or without 100μg/mL CHX for the indicated times. Relative GPD1L protein levels (GPD1L: β-Actin) are shown in (**g**). (**h**) Immunoblot analysis of the indicated proteins in HCCLM3 cells treated with or without 10μM MG132, CQ. (**i**) GPD1L ubiquitination sites predicted by GPS-uber database. (**j**) Immunoblot analysis of HA beads pull-down products and input derived from 293T cells transfected with FLAG-VCP and HA-tagged constructed mutants. (**k**) Immunoblot analysis of FLAG beads pull-down products and input derived from 293T cells co-transfected with the indicated plasmids for 48h. (**l,m**) Immunoblot analysis of HA beads pull-down products and input derived from HCCLM3/293T cells co-transfected with the indicated plasmids for 48h. (**n**) 293T cell lysates were subject to immunoprecipitation with control IgG, anti-VCP antibodies. The immunoprecipitates were then blotted. (**o**) Immunofluorescence analysis to detect colocalization of VCP and GPD1L in Hepa1-6 cells. (**p**) Immunoblot analysis of the indicated proteins in HCCLM3 cells transfected with the indicated plasmids. (**q**) Cytotoxicity assessed by measuring lactate dehydrogenase (LDHA) release from Hepa1-6-OVA cells (n = 3). (**r**) Extracellular G3P levels in Hepa1-6-OVA cells (n = 3). Data are presented as mean values ± SD. Statistical significance was determined using two sided t-tests, **P* < 0.05, ***P*< 0.01, and ****P* < 0.001.


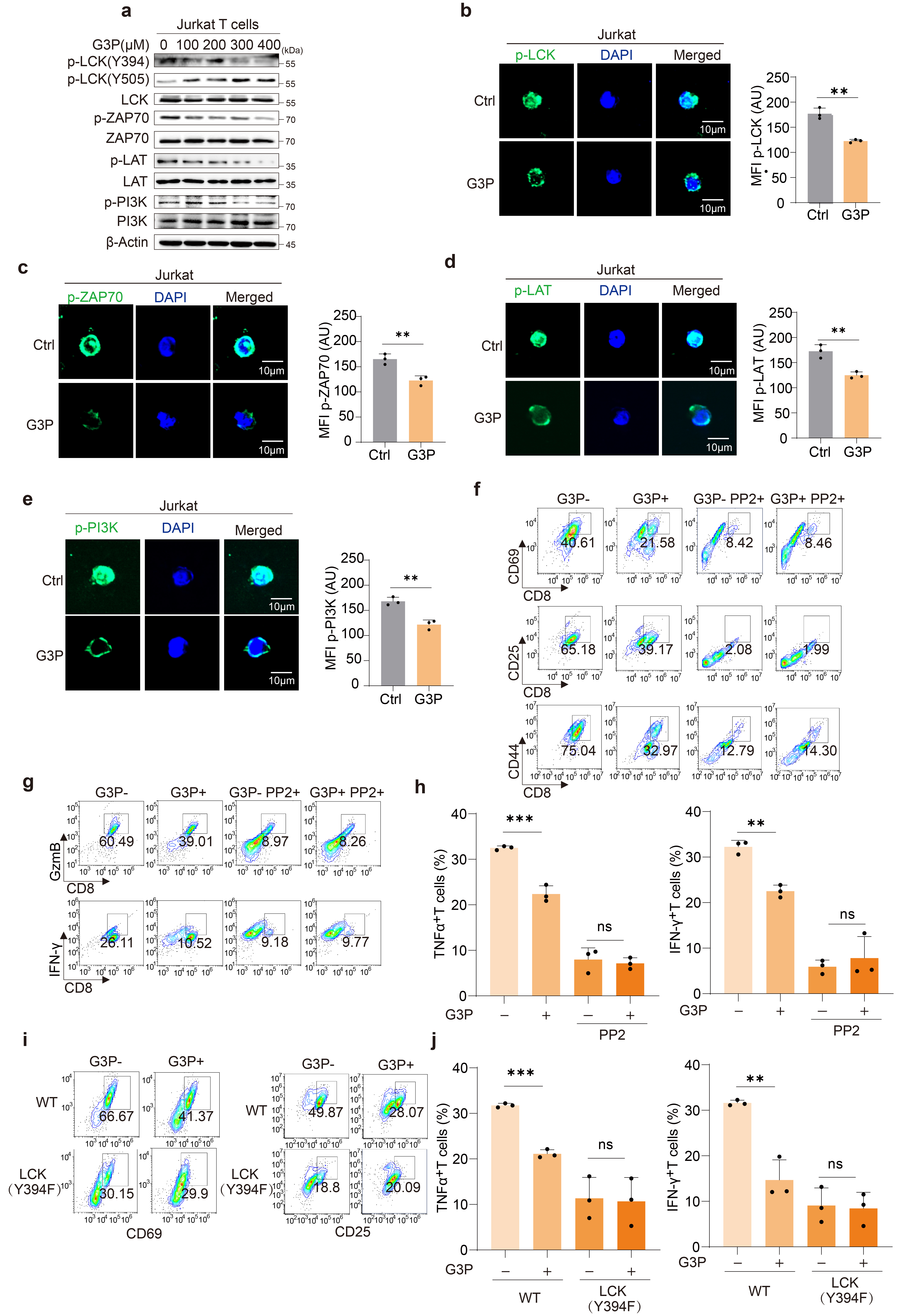


Figure. S6.

G3P inhibits the LCK signaling. (**a**) Immunoblot analysis of the indicated proteins in Jurkat T cells treated with gradient concentrations of G3P. (**b-e**) Left, Jurkat T cells treated with G3P or untreated (Ctrl) followed by stimulation were subjected to immunostaining and confocal microscopic imaging. Right, quantification of the mean fluorescence intensity. (**f,g**) Related to Fig. 5i,j. Representative contour plot of CD8^+^T cells treated with G3P and/or PP2 was measured by flow cytometry (n = 3). (**h**) The percentage of cytokines produced by Jurkat T cells treated with G3P and/or PP2 was measured by flow cytometry (n = 3). (**i**) Related to Fig. 5k. Representative contour plot of Jurkat T cells was measured by flow cytometry (n = 3). (**j**) Jurkat T cells expressing WT–LCK or the LCK(Y394F) mutant were treated with G3P or untreated. The percentage of cytokines was determined by flow cytometry (n = 3). Data are presented as mean values ± SD. Statistical significance was determined using two sided t-tests, **P* < 0.05, ***P*< 0.01, and ****P* < 0.001.


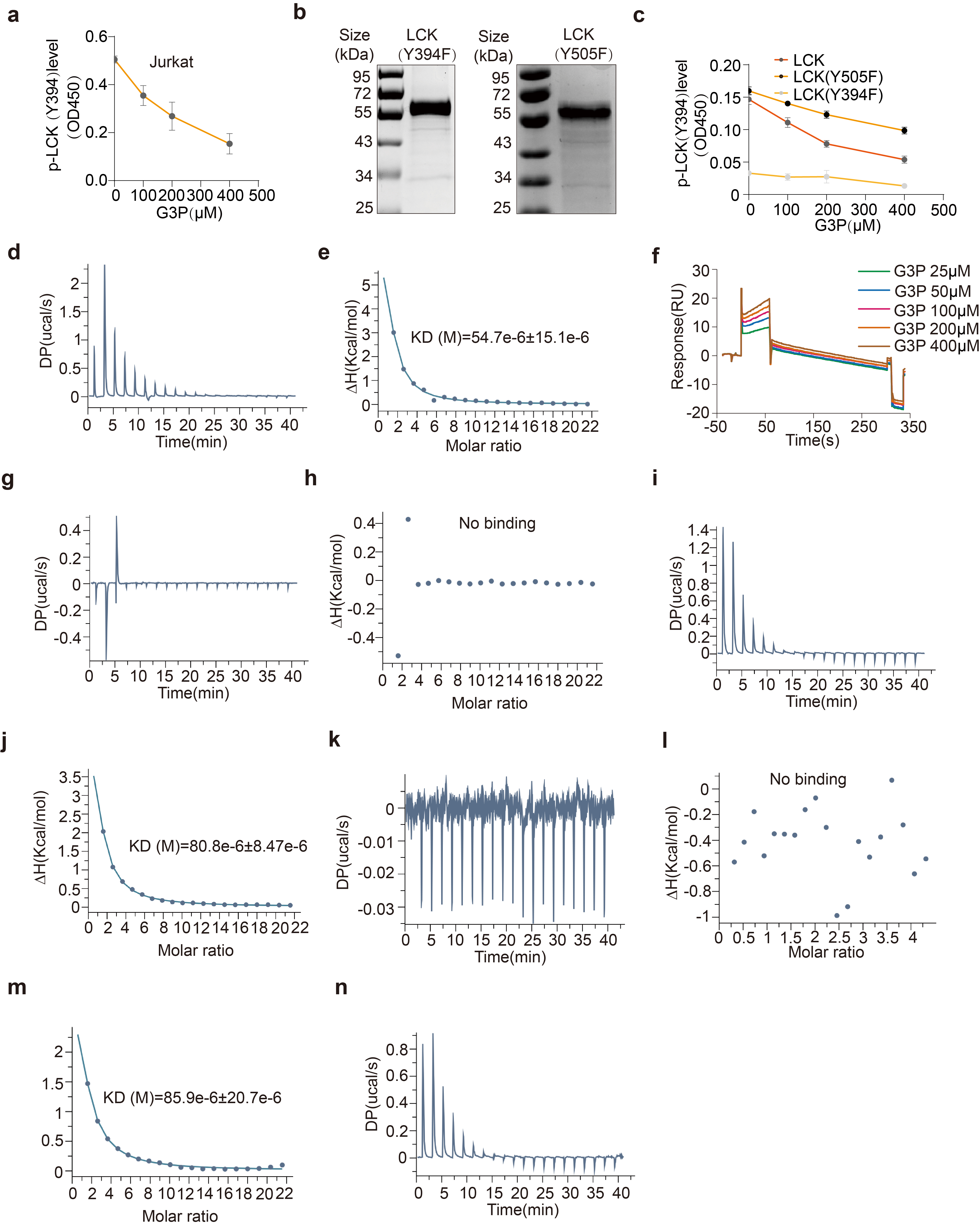


Figure. S7.

G3P binds directly to LCK. (**a**) The p-LCK(Y394) levels of Jurkat T cells treated with gradient concentrations of G3P. (**b**) Purified LCK protein mutants were analysed by SDS–PAGE, followed by Coomassie blue staining. (**c**) In vitro kinase assay for LCK and its mutants in the presence of increasing amounts of G3P. Kinase activity is expressed as relative values to phosphorylation of LCK(Y394). (**d,e**) Related to Fig. 6e. ITC measurement of the interaction between G3P and the purified LCK. (**f**) Related to Fig. 6j. Real-time sensor map of the interaction between G3P and the purified region. (**g,h**) Related to Fig. 6h. ITC measurement of the interaction between G3P and the purified region. (**i,j**) Related to Fig. 6j. ITC measurement of the interaction between G3P and the purified region. (**k,l**) Related to Fig. 6l. ITC measurement of the interaction between G3P and the purified region. (**m,n**) Related to Fig. 6n. ITC measurement of the interaction between G3P and the purified region.


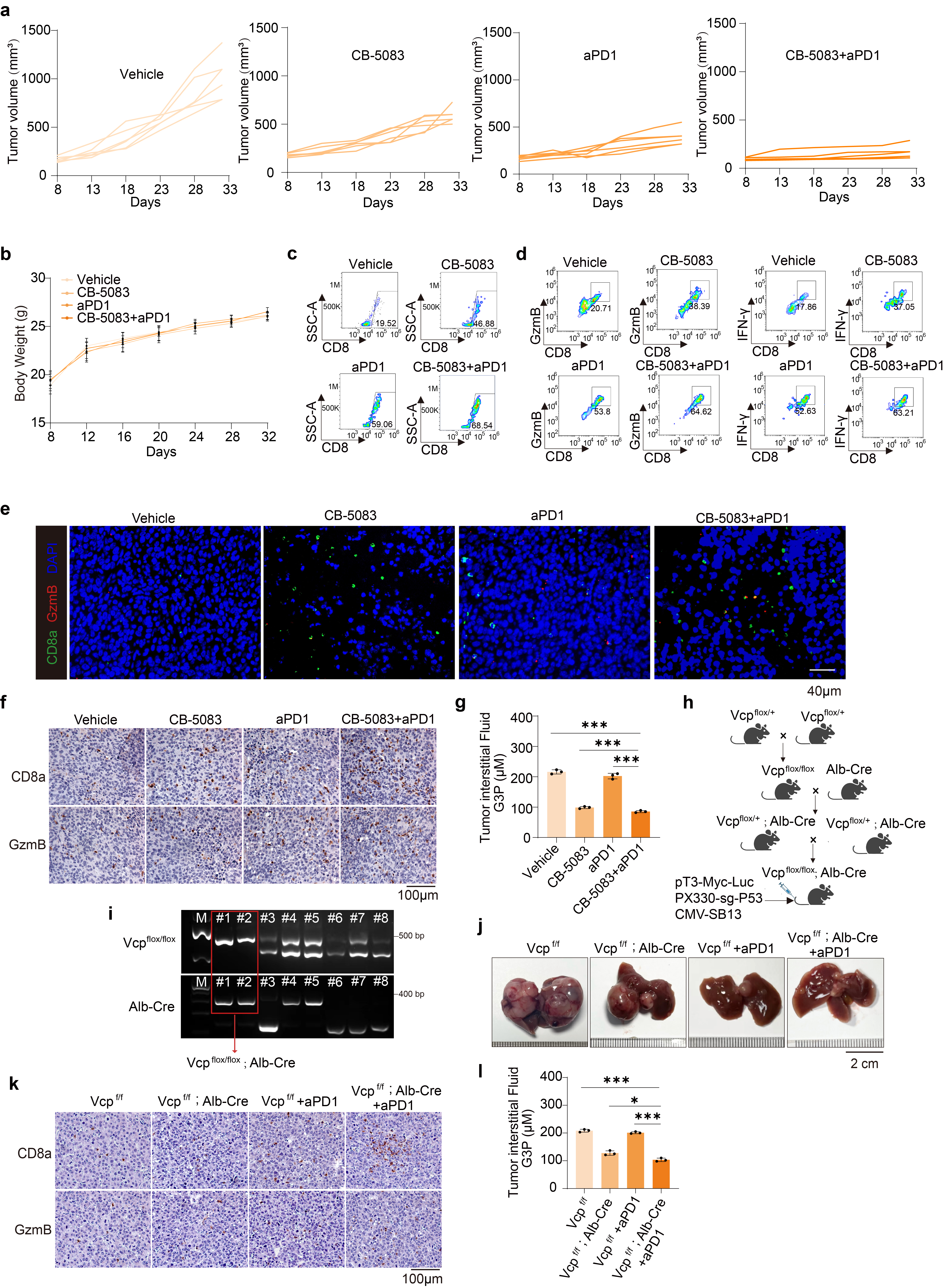


Figure. S8.

VCP inhibition or loss plus anti-PD1 can improve the immune microenvironment. (**a**) Individual tumor growth curves for vehicle, CB-5083, aPD1, or both.(n = 6/group). (**b**) Body weight was measured every 4 days. (**c**) Related to Fig. 7d. Representative contour plots of percentage of CD8^+^T cells in Hepa1-6 tumors were determined by flow cytometry. (**d**) Related to Fig. 7e. Representative contour plots of percentage of cytokines produced by CD8^+^T cells in Hepa1-6 tumors were determined by flow cytometry. (**e,f**) Hepa1-6 tumors sections were stained with multiple immunofluorescence and immunohistochemistry. (**g**) G3P levels in interstitial fluid of Hepa1-6 tumors (n = 3). (**h**) Schematic of Vcp conditional knockout mouse. Schematic created with BioRender.com. (**i**) The genotype identification of Vcp^f/f^; Alb-Cre mice. (**j**) Representative images of livers from Fig. 7g. (**k**) Spontaneous tumor sections of conditional knockout mice were stained with immunohistochemistry. (**l**) G3P levels in interstitial fluid of spontaneous tumor sections of conditional knockout mice (n = 3). Data are presented as mean values ± SD. Statistical significance was determined using two sided t-tests, **P* < 0.05, ***P*< 0.01, and ****P* < 0.001.

**Table S1. shRNA sequences and primers used in this study.**

| **shRNA sequences** | **Sequence (5'-3')** | |
| --- | --- | --- |
| shVCP-1 | CGCATTGTATCACAGTTGT | |
| shVCP-2 | CTGTGGAGCACCCAGACAA | |
| shVCP-3 | AGATGGATCTCATTGACCT | |
| M-shVcp | GCCGTCTAGATCAGCTCATTT | |
| shGPD1L-1 | ACGAAAGCAGACCAGTTCA | |
| **Primers used in qRT-PCR** | | |
| **Name** | **Sequence (5'-3')** | |
|  | **Forward** | **Reverse** |
| M-β-Actin | GGCTGTATTCCCCTCCATCG | CCAGTTGGTAACAATGCCATGT |
| M-Vcp | GGTGAGTCTGAGAGCAACCT | AACAACTGAGACACGATGCG |
| M-Gpd1l | TCTTCTGTAAGGGCCAGGTG | TCTCCAGCTCTTCGATGGTC |
| M-Gpd1 | GAGGTGGCTGAGGAGAAGTT | CTCCACTGTGTCCACCTCTT |
| **Primers used in Mouse Genotyping** | | |
| **Name** | **Sequence (5'-3')** | |
| Vcp^f/f^ | GCAGTTTCTGCCTTCATTGTGG | |
|  | GTTTGATTTACAGGAGTGCCTCAAG | |
| Alb-cre | TGCAAACATCACATGCACAC | |
|  | TTGGCCCCTTACCATAACTG | |
|  | GAAGCAGAAGCTTAGGAAGATGG | |

**Table S2. Clinical information of HCC patients.**

| Patients | Gender | Age | Pathology |
| --- | --- | --- | --- |
| P1 | Male | 66 | Medium-differentiated HCC of the liver, size 4cm*4cm*3cm. |
| P2 | Male | 74 | Medium to poorly differentiated HCC of the liver, size 3.5cm*2.5cm*2cm. |
| P3 | Male | 52 | Medium to poorly differentiated HCC of the liver, size 11cm*8cm*7cm. |
| P4 | Male | 60 | Medium to poorly differentiated HCC of the liver, size 7.5cm*7.5cm*4.5cm and 5.0cm*4.0cm*4.0cm. |
| P5 | Male | 49 | Medium to poorly differentiated HCC of the liver, size 3.5cm*3.0cm*1.5cm. |
| P6 | Male | 51 | Medium-differentiated HCC of the liver, size 8.5cm*4cm*3cm. |
| P7 | Male | 49 | Medium-differentiated HCC of the liver, size 4.2cm*4.1cm*2.9cm. |
| P8 | Male | 54 | Medium to highly differentiated HCC of the liver, size 6.5cm*5.0cm*4.5cm. |
| P9 | Male | 63 | Medium-differentiated HCC of the liver, size 3cm*1.5cm*1.2cm. |
| P10 | Male | 52 | Medium-differentiated HCC of the liver, size 7cm*6cm*5.5cm. |
| P11 | Female | 67 | Medium to poorly differentiated HCC of the liver, size 2.5cm*2.5cm*1.5cm. |
| P12 | Female | 51 | Poorly differentiated HCC of the liver, size 2.0cm*1.5cm*0.6cm. |
| P13 | Male | 69 | Medium-differentiated HCC of the liver, size 14.0cm*9.0cm*8.0cm. |
| P14 | Male | 71 | Medium-differentiated HCC of the liver, size 12cm*9cm*7cm. |
| P15 | Female | 70 | Medium-differentiated HCC of the liver, size 8.5cm*8.0cm*6.0cm. |
| P16 | Male | 57 | Medium to poorly differentiated HCC of the liver, size 6.5cm*5.0cm*4.5cm. |
| P17 | Male | 57 | Medium-differentiated HCC of the liver, size 14.8cm*7.5cm*7.4cm. |
| P18 | Male | 61 | Medium-differentiated HCC of the liver, size 5.0cm*5.0cm*4.0cm. |
| P19 | Male | 34 | Medium to poorly differentiated HCC of the liver, size 4.5cm*4.5cm*3.2cm. |
| P20 | Male | 55 | Medium-differentiated HCC of the liver, size 7.0cm*5.5cm*4.5cm. |
