# Supplementary material for: VCP downstream metabolite glycerol-3-phosphate (G3P) inhibits CD8+T cells function in the HCC microenvironment
Source: Signal Transduct Target Ther. 2025 Jan 24;10:26. doi: 10.1038/s41392-024-02120-8 (PMC11758394; doi:10.1038/s41392-024-02120-8)

Original WB images

VCP downstream metabolite glycerol-3-phosphate (G3P) inhibits $CD8^+$ T cells function in the HCC microenvironment

Cheng Cheng, Qingrui Zha, Linmao Sun, Tianming Cui, Xinyu Guo, Changjian Xing, Zhengxiang Chen, Changyong Ji, Shuhang Liang, Shengwei Tao, Junhui Chu, Chenghui Wu, Qi Chu, Xuetian Gu, Ning Zhang, Yumin Fu, Shumin Deng, Yitong Zhu, Jiabei Wang, Yao Liu, Lianxin Liu,

The bands for the representative images used in the manuscript had been marked in red box.

Fig. 4b:VCP

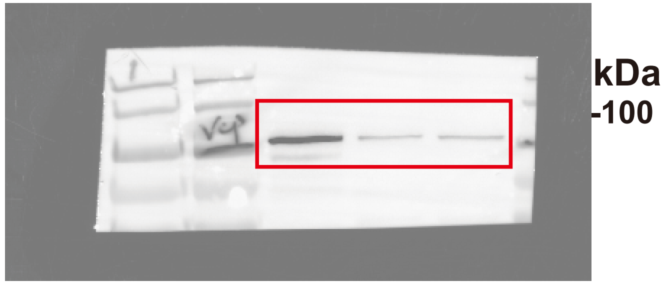

Fig. 4c:FLAG-VCP

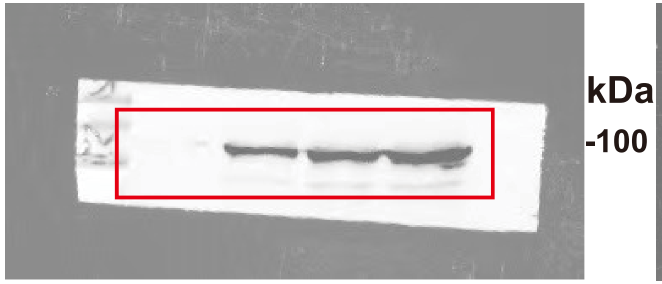

Fig. 4d:VCP

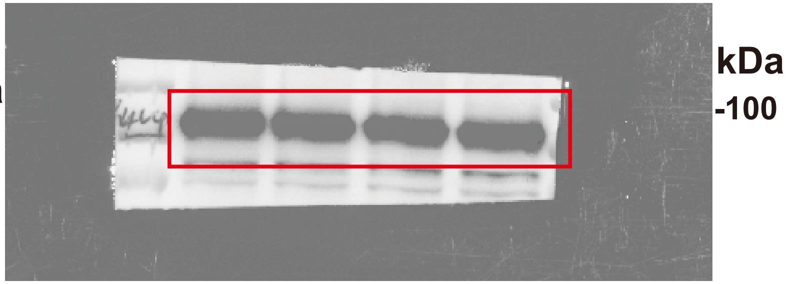

Fig. 4b:GPD1L

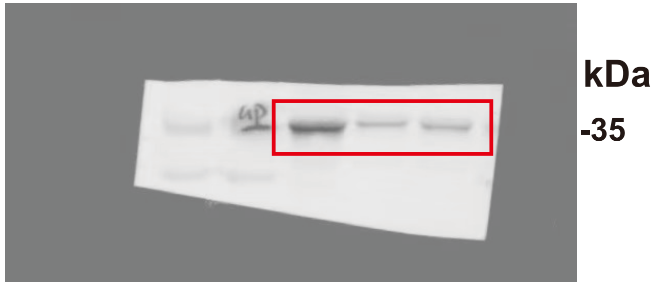

Fig. 4c:GPD1L

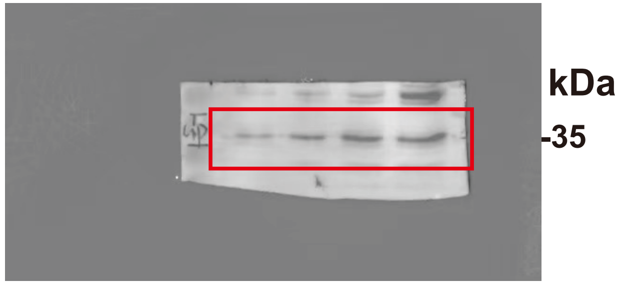

Fig. 4d:SQSTM1

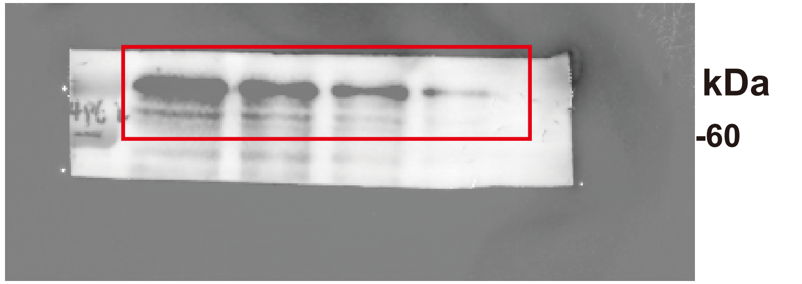

Fig. 4b:GPD1

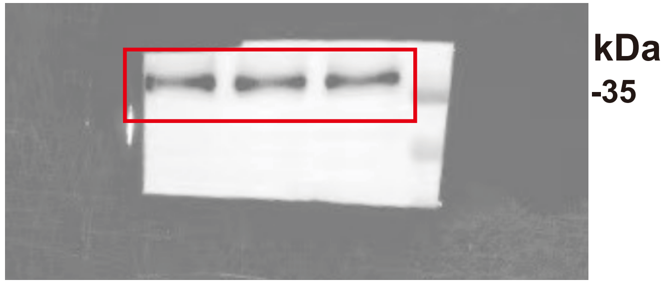

Fig. 4c:β-Actin

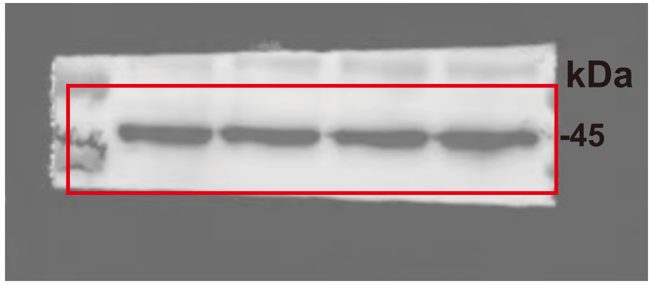

Fig. 4d:GPD1L

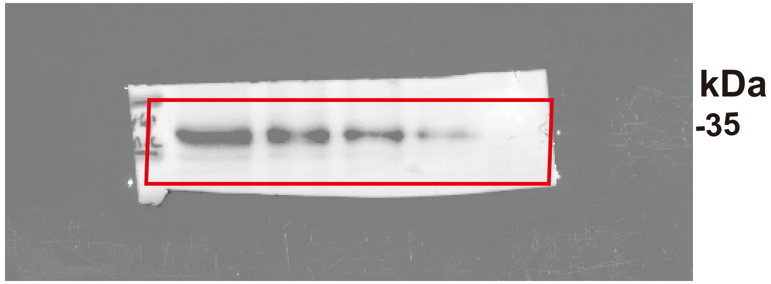

Fig. 4b:β-Actin

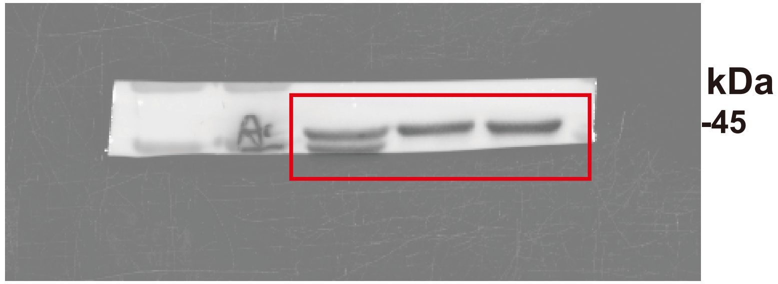

Fig. 4d:β-Actin

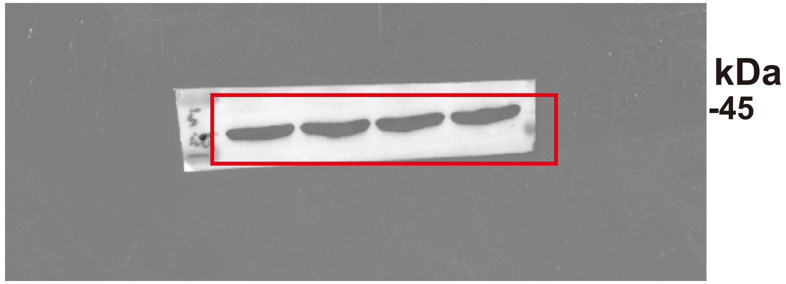

Fig. 4e:VCP

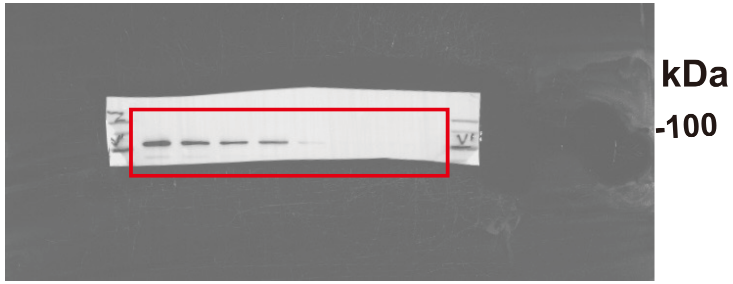

Fig. 4e:GPD1L

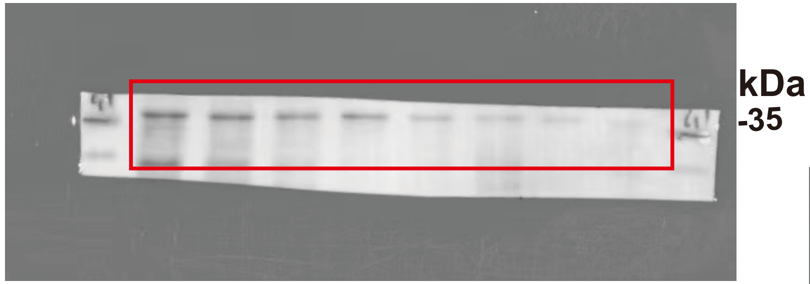

Fig. 4e:β-Actin

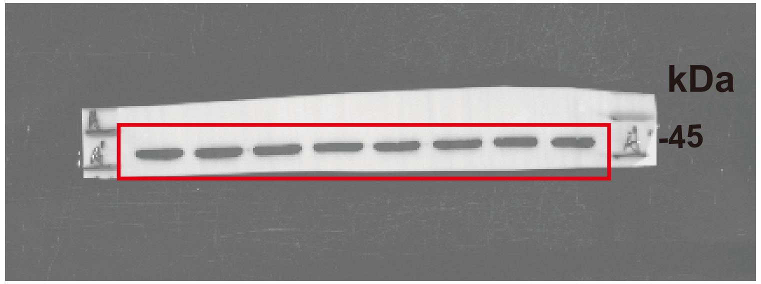

Fig. 4g:HA-IP-His

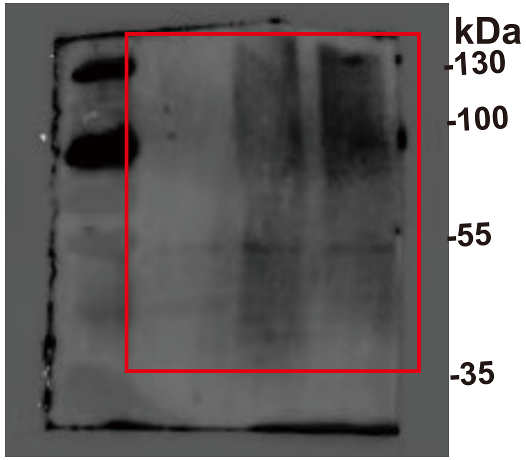

Fig. 4g:HA-IP-HA

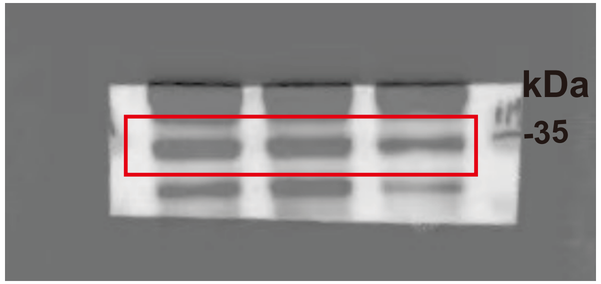

Fig. 4g:Input-HA

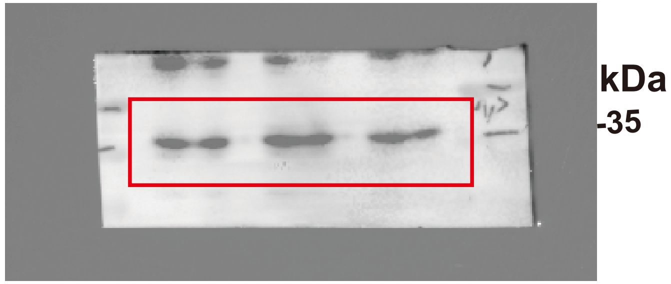

Fig. 4g:Input-VCP

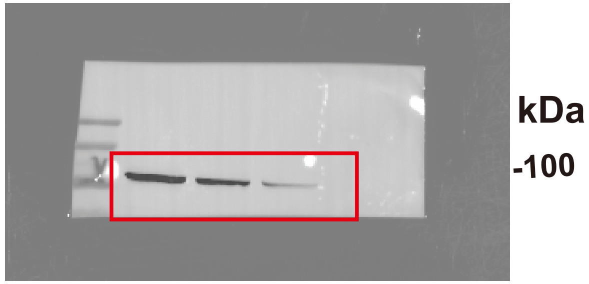

Fig. 4g:Input-β-Actin

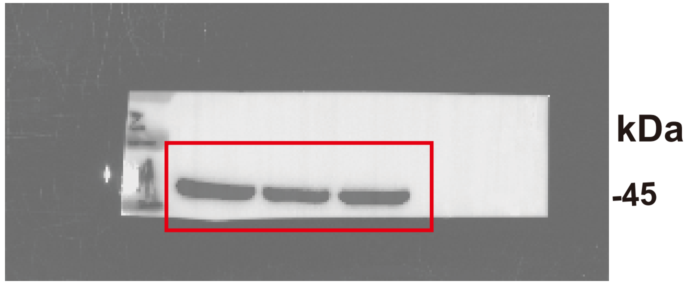

Fig. 4h:HA-IP-His

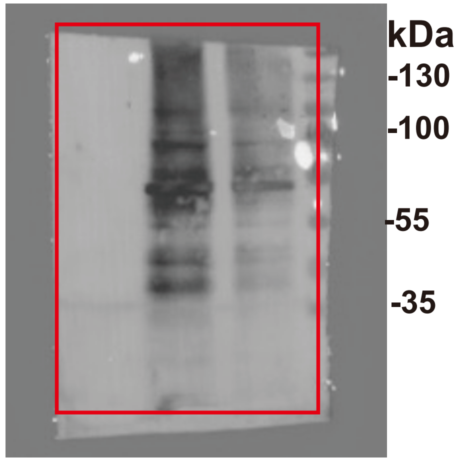

Fig. 4h:HA-IP-HA

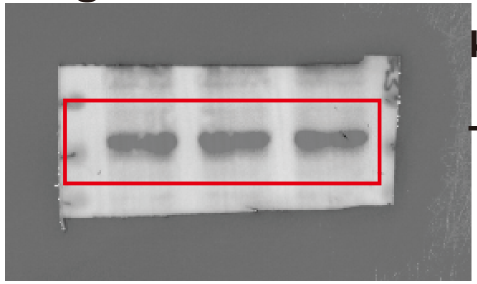

Fig. 4h:Input-HA

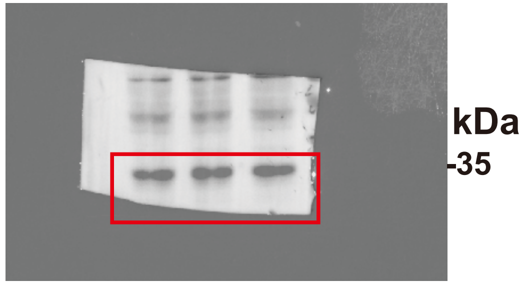

Fig. 4h:Input-VCP

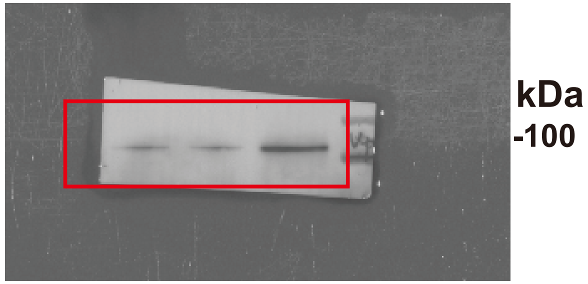

Fig. 4h:Input-β-Actin

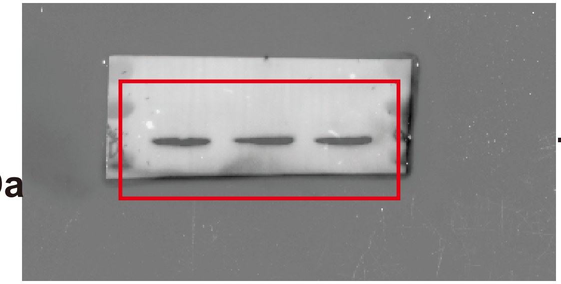

Fig. 4i:FLAG-IP-HA

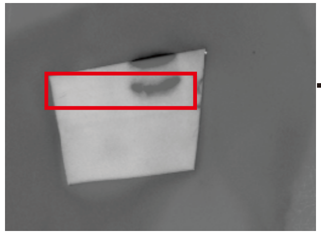

Fig. 4i:FLAG-IP-FLAG

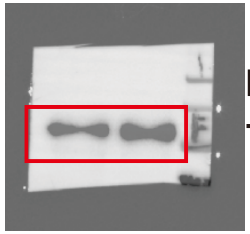

Fig. 4i:Input-HA

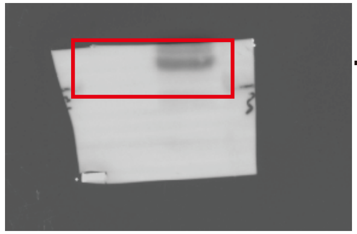

Fig. 4i:Input-FLAG

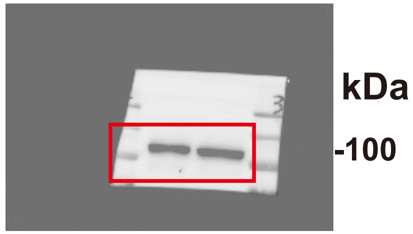

Fig. 4i:Input-β-Actin

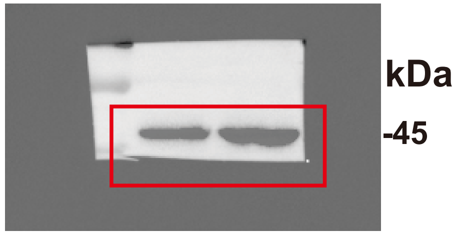

Fig. 4j:VCP

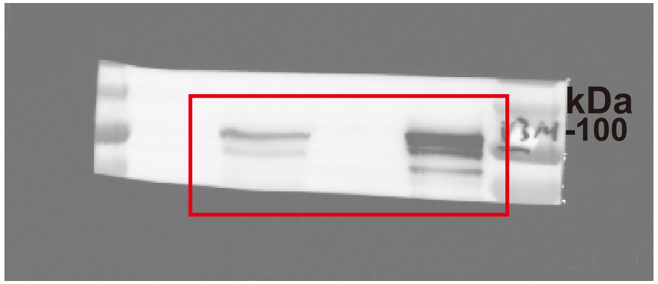

Fig. 4k:His

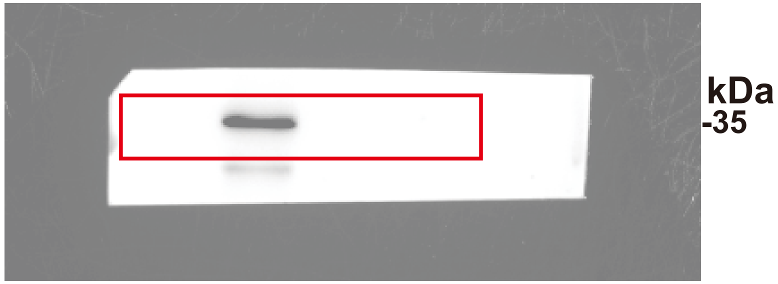

Fig. 4o:FLAG-IP-HA

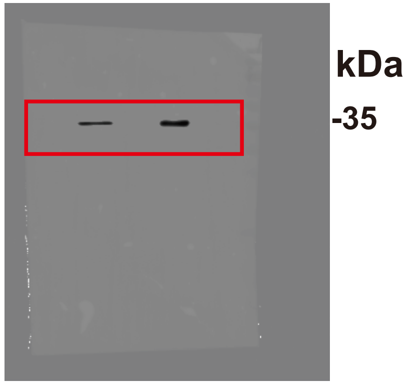

Fig. 4j:GPD1L

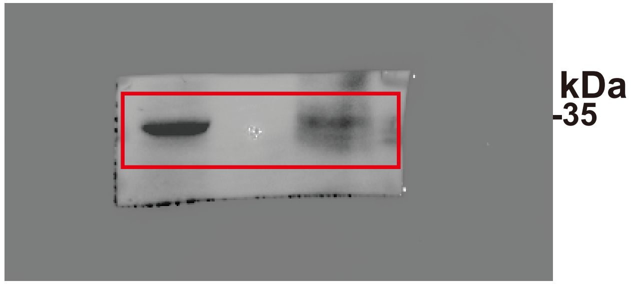

Fig. 4k:GST

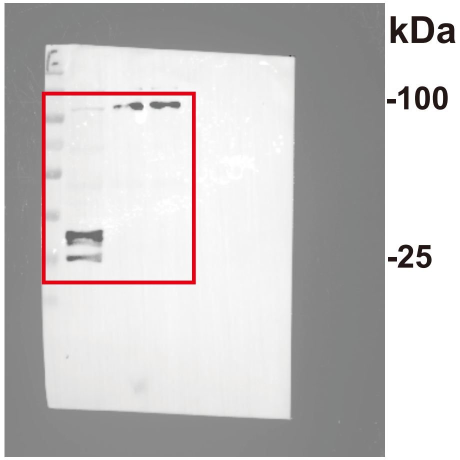

Fig. 4o:input-HA

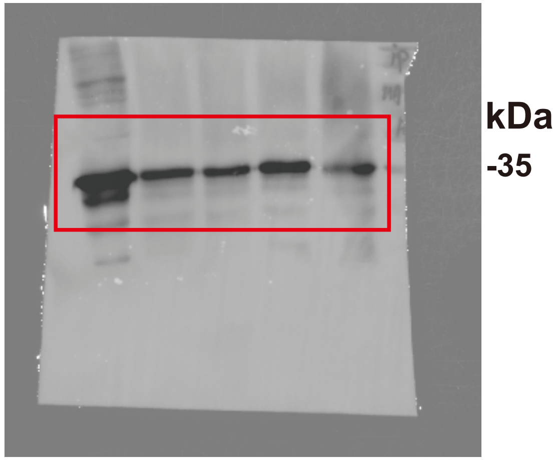

Fig. 4o:input-FLAG

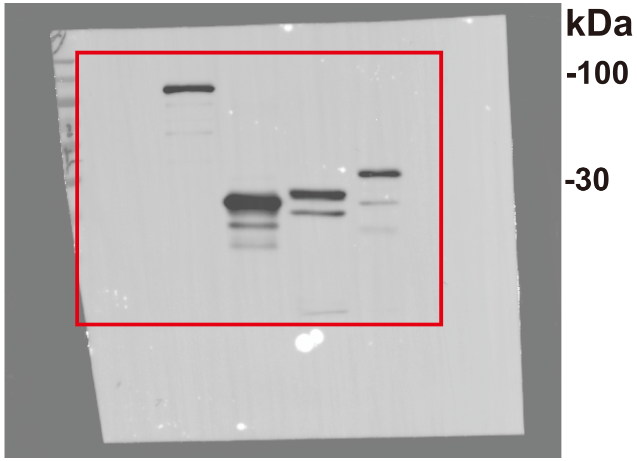

Fig. 4p:HA-IP-FLAG

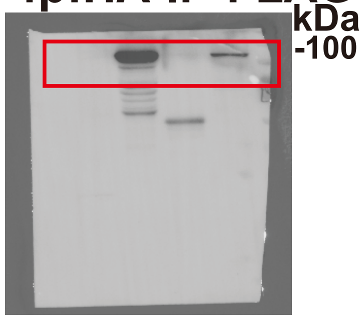

Fig. 4p:input-FLAG

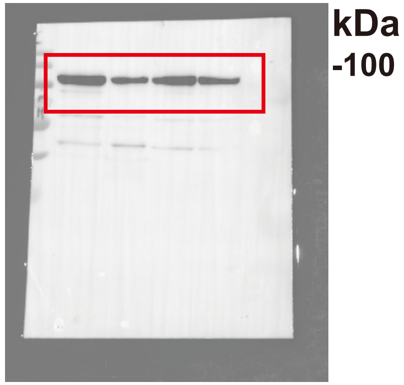

Fig. 4p:input-HA

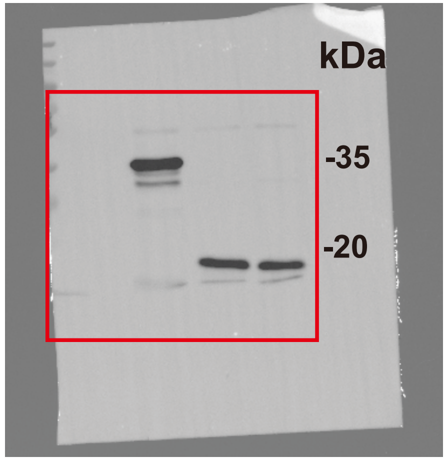

Fig. 5b:Hepa1-6-VCP

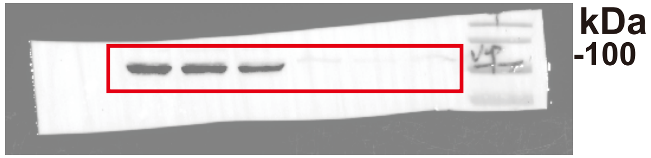

Fig. 5b:Hepa1-6-β-Actin

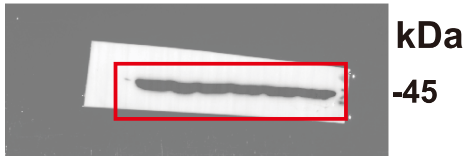

Fig. 5b:CD8-p-LCK(Y394)

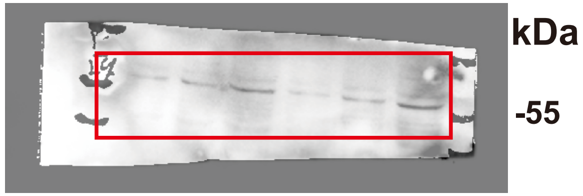

Fig. 5b:CD8-LCK

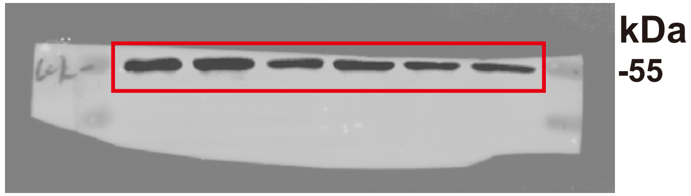

Fig. 5b:CD8-p-ZAP70

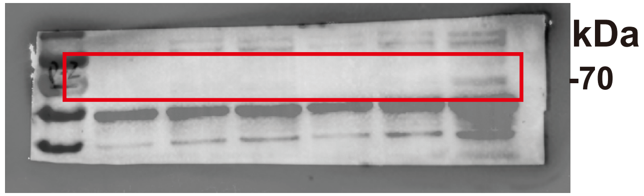

Fig. 5b:CD8-ZAP70

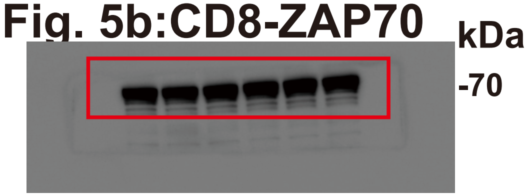

Fig. 5b:CD8-p-LAT

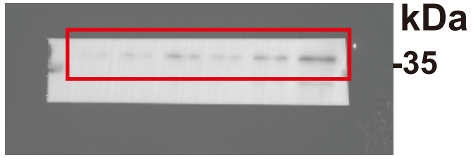

Fig. 5b:CD8-LAT

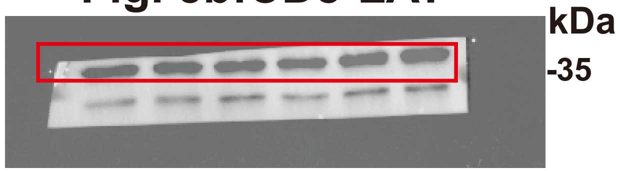

Fig. 5b:CD8-p-PI3K

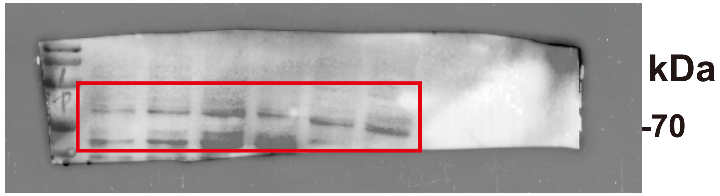

Fig. 5b:CD8-PI3K

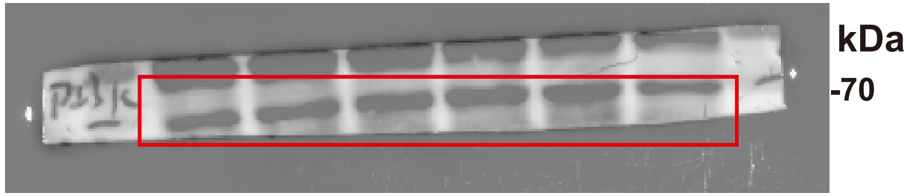

Fig. 5b:CD8-β-Actin

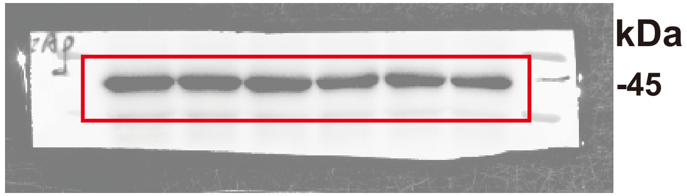

Fig. 5c:p-LCK(Y394)

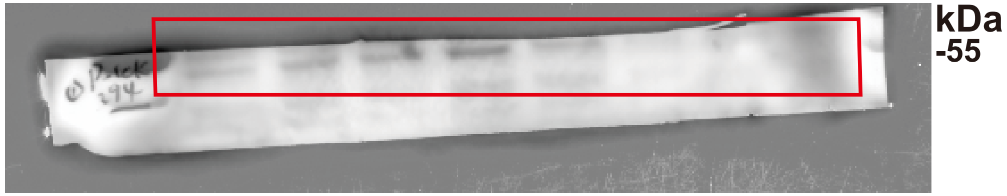

Fig. 5c:p-LCK(Y505)

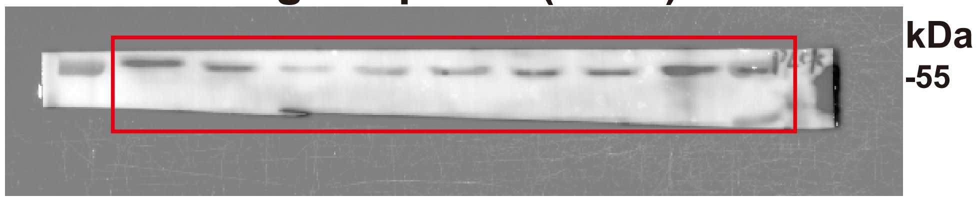

Fig. 5c:LCK

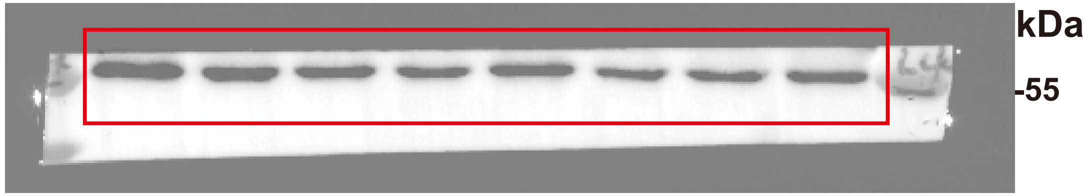

Fig. 5c:p-ZAP70

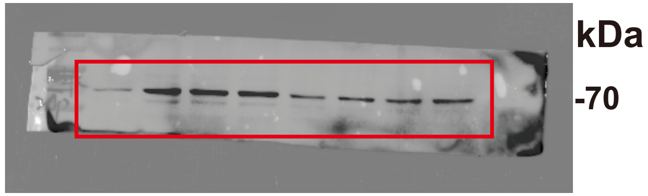

Fig. 5c:ZAP70

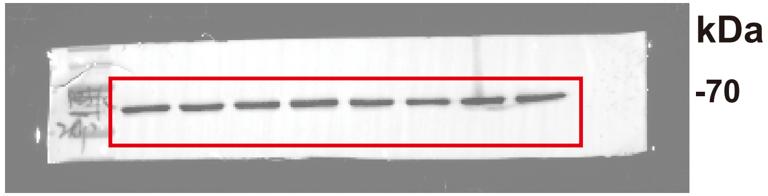

Fig. 5c:p-LAT

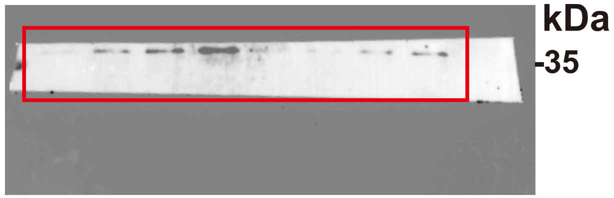

Fig. 5c:LAT

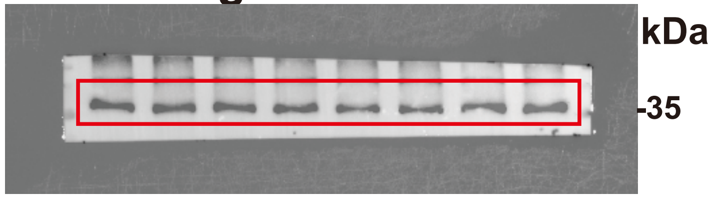

Fig. 5c:p-PI3K

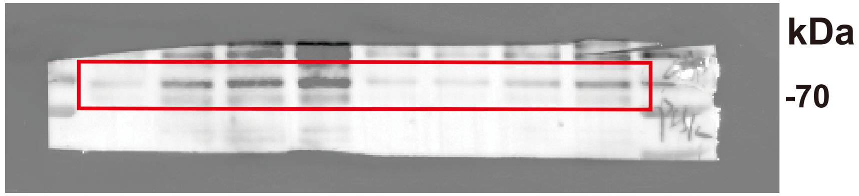

Fig. 5c:PI3K

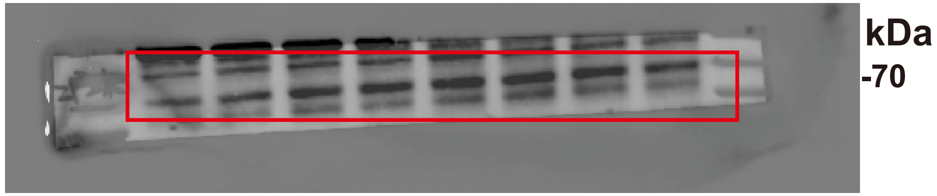

Fig. 5c:β-Actin

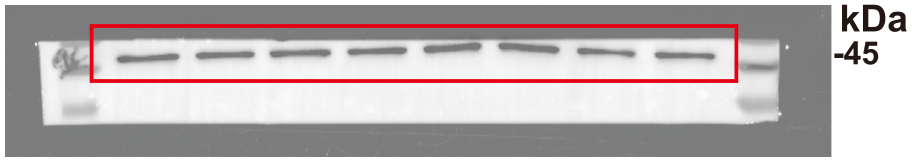

Fig. 5h:p-LCK(Y394)

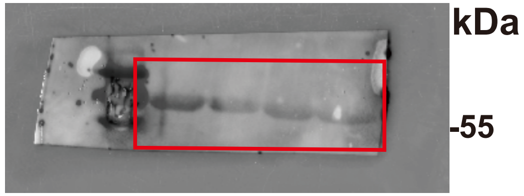

Fig. 5h:p-LCK(Y505)

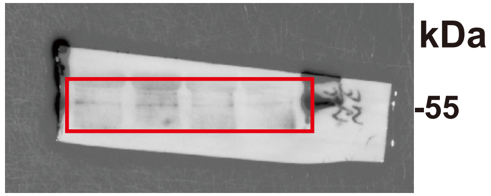

Fig. 5h:LCK

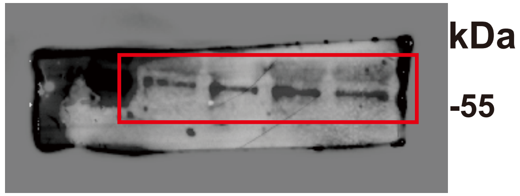

Fig. 5h:p-ZAP70

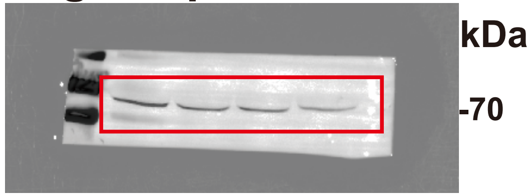

Fig. 5h:ZAP70

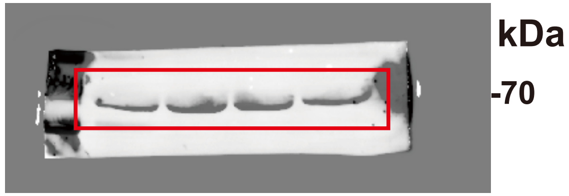

Fig. 5h:p-LAT

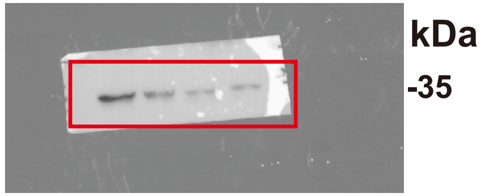

Fig. 5h:LAT

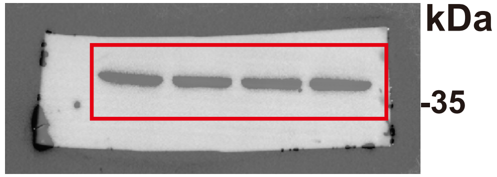

Fig. 5h:p-PI3K

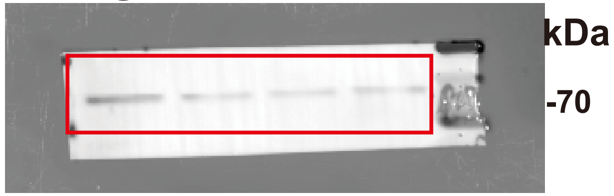

Fig. 5h:PI3K

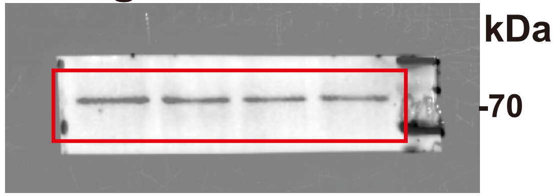

Fig. 5h:β-Actin

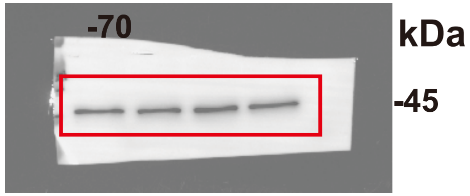

Fig. 6b:p-LCK(Y394)

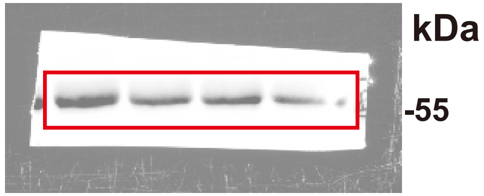

Fig. 6b:p-LCK(Y505)

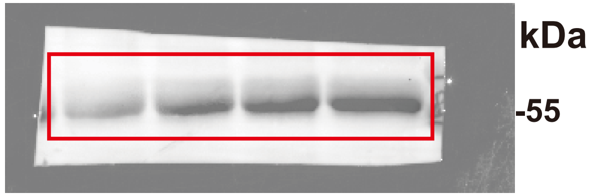

Fig. 6b:LCK

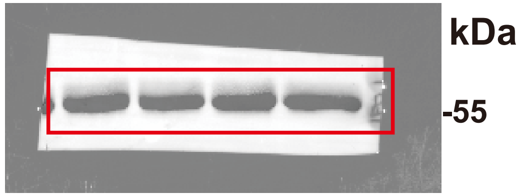

Fig. 6c:purified LCK-p-LCK(Y505)

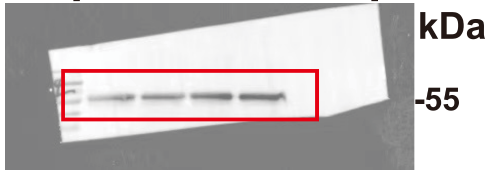

Fig. 6c:purified LCK-p-LCK(Y394)

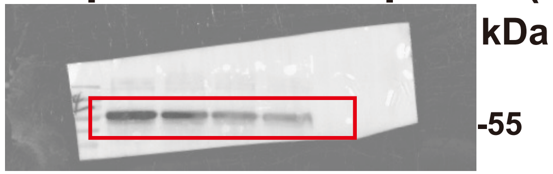

Fig. 6c:purified LCK-LCK

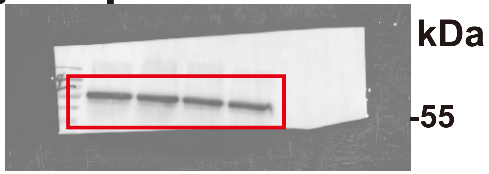

Fig. 6c:purified LCKY394-p-LCK(Y505)

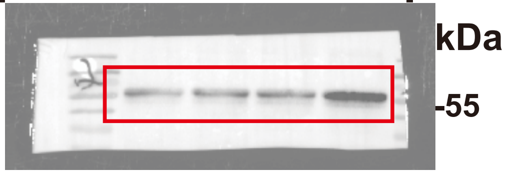

Fig. 6c:purified LCKY394-p-LCK(Y394)

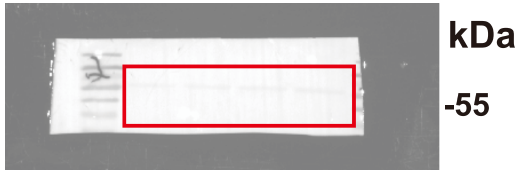

Fig. 6c:purified LCKY394-LCK

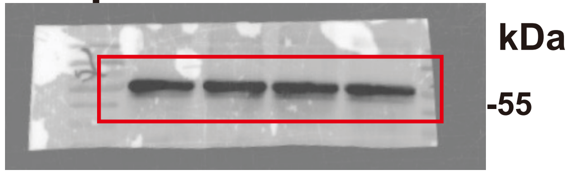

Fig. 6c:2-purified LCK-p-LCK(Y505)

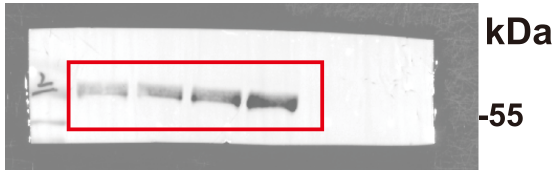

Fig. 6c:2-purified LCK-p-LCK(Y394)

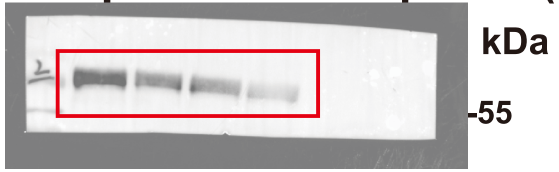

Fig. 6c:2-purified LCK-LCK

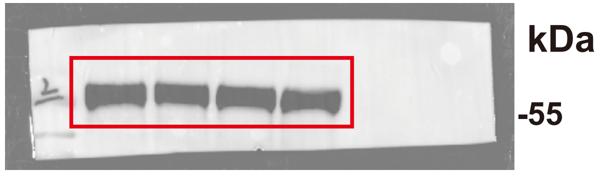

Fig. 6c:purified LCKY505-p-LCK(Y505)

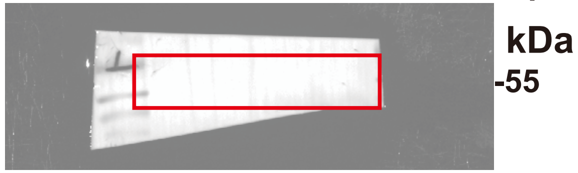

Fig. 6c:purified LCKY505-p-LCK(Y394)

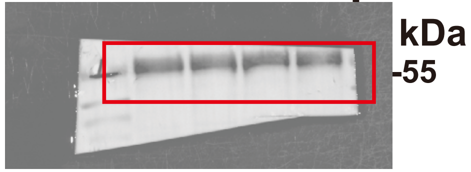

Fig. 6c:purified LCKY505-LCK

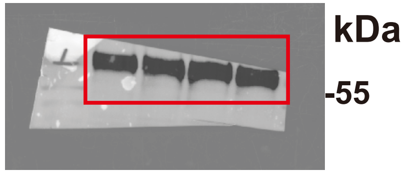

Fig. 6d:purified LCK

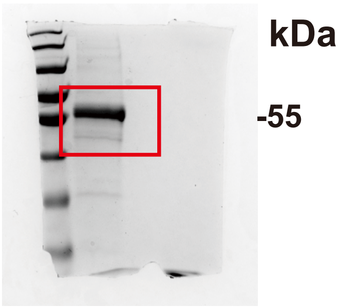

Fig. 6h:purified LCK(60-240AAs)

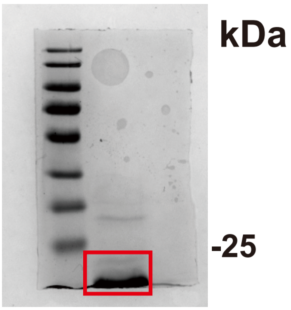

Fig. 6j:purified LCK(240-509AAs)

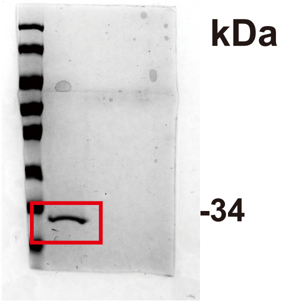

Fig. 6l:purified LCK(230-390AAs)

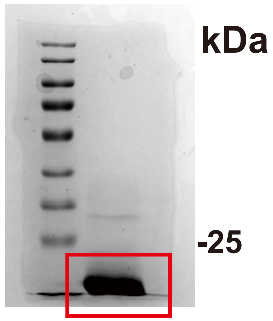

Fig. 6n:purified LCK(330-509AAs)

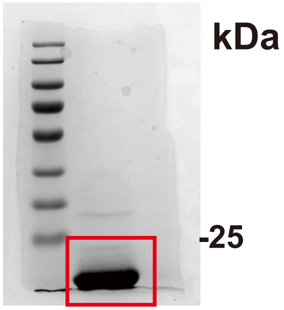

Suppl Fig. 1b:Hepa1-6 tumors-VCP

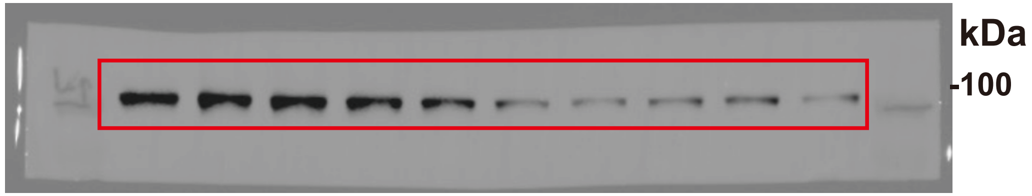

Suppl Fig. 1b:Hepa1-6 tumors-β-Actin

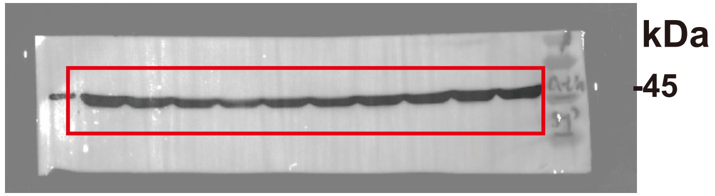

Suppl Fig. 1b:Spontaneous tumors-VCP

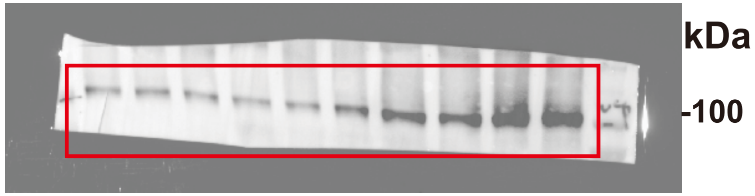

Suppl Fig. 1b:Spontaneous tumors-β-Actin

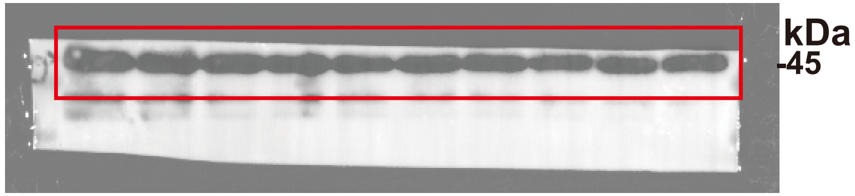

Suppl Fig. 4a:GPD1L

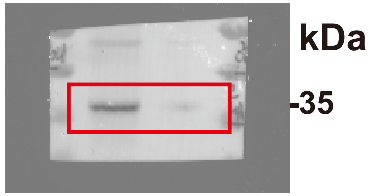

Suppl Fig. 4a:β-Actin

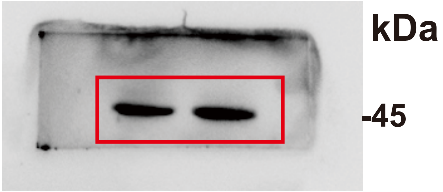

Suppl Fig. 4a:GPD1

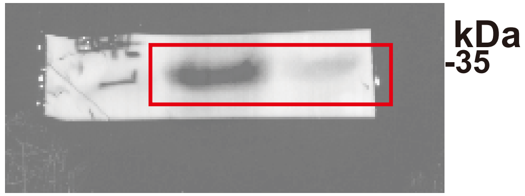

Suppl Fig. 4a:2-β-Actin

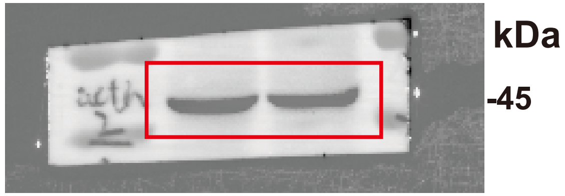

Suppl Fig. 4d:VCP

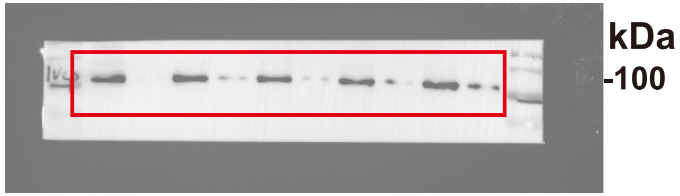

Suppl Fig. 4e:VCP

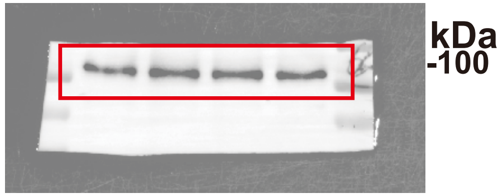

Suppl Fig. 4f:FLAG

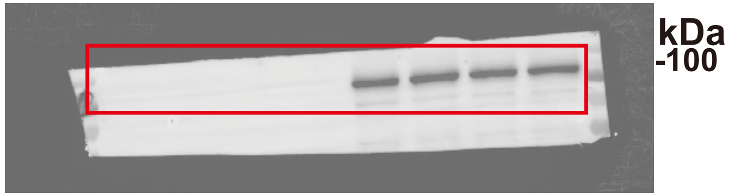

Suppl Fig. 4d:GPD1L

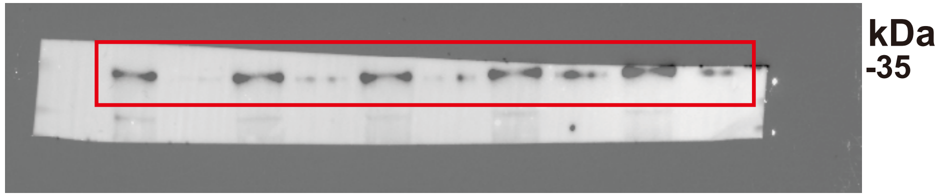

Suppl Fig. 4e:SQSTM1

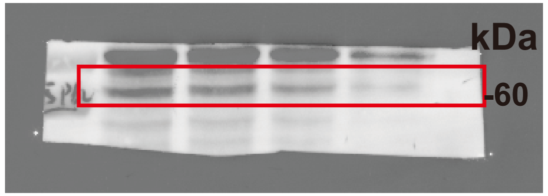

Suppl Fig. 4f:GPD1L

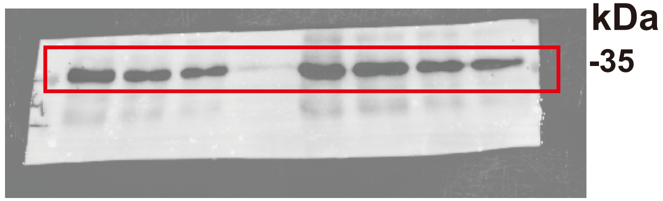

Suppl Fig. 4d:β-Actin

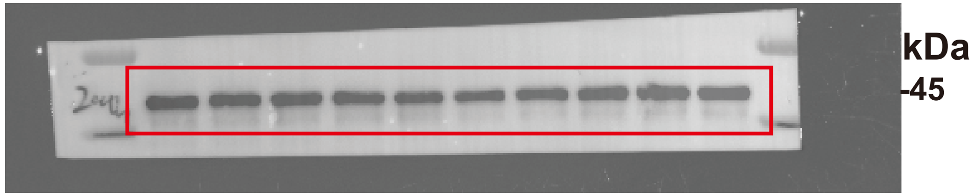

Suppl Fig. 4e:GPD1L

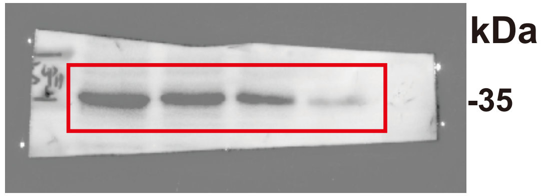

Suppl Fig. 4f:β-Actin

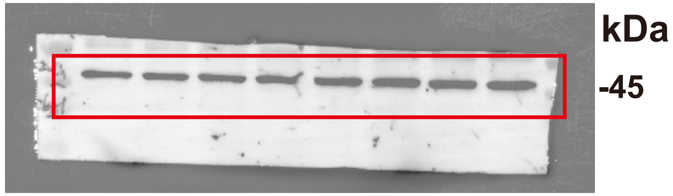

Suppl Fig. 4e:β-Actin

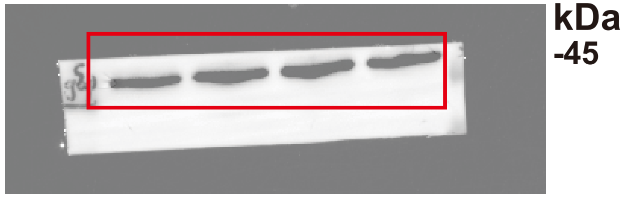

Suppl Fig. 4h:GPD1L

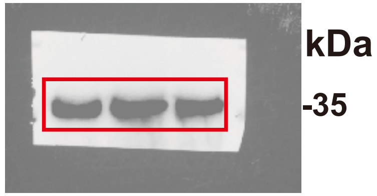

Suppl Fig. 4h:β-Actin

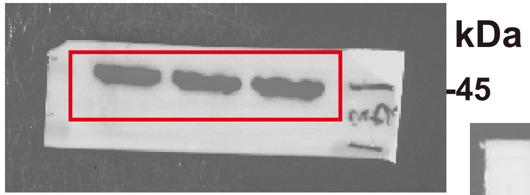

Suppl Fig. 4j:His

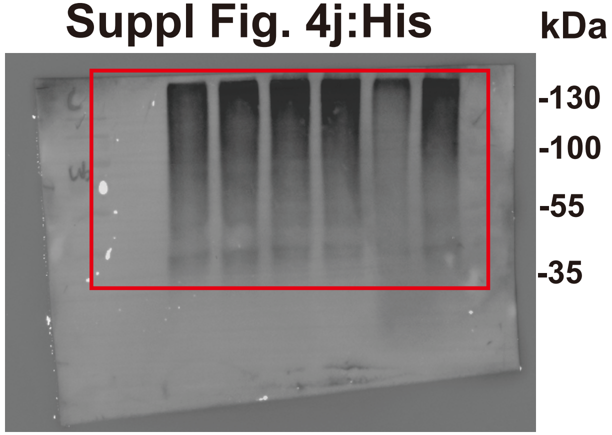

Suppl Fig. 4h:IP-HA

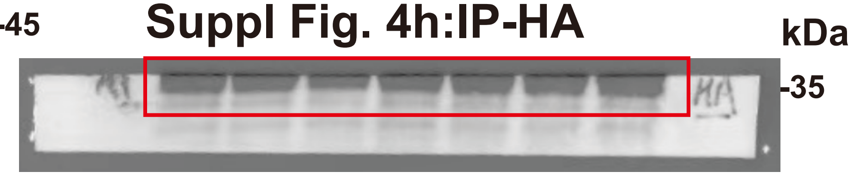

Suppl Fig. 4h:input-HA

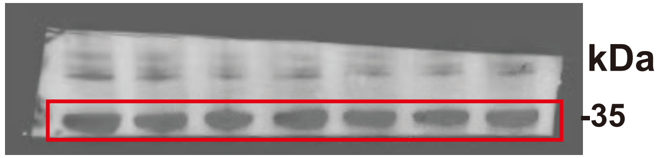

Suppl Fig. 4h:input-FLAG

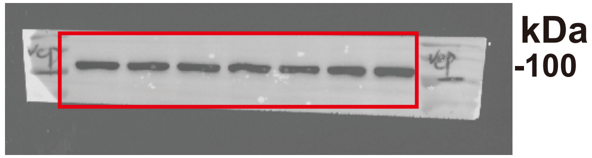

Suppl Fig. 4h:input-β-Actin

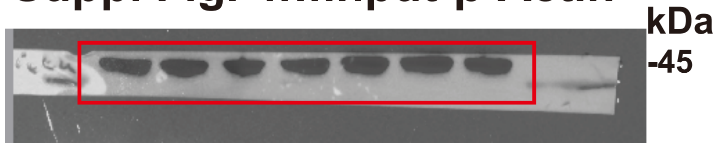

Suppl Fig. 4k:IP-HA

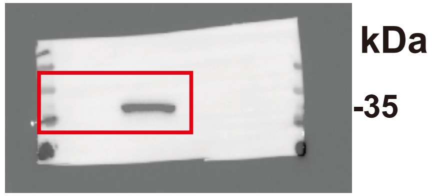

Suppl Fig. 4k:IP-FLAG

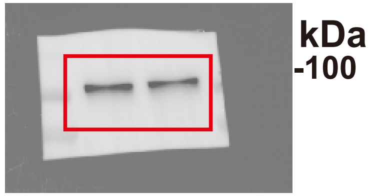

Suppl Fig. 4k:input-HA

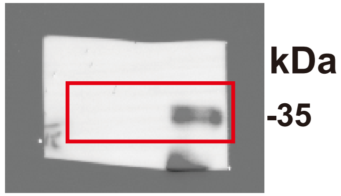

Suppl Fig. 4k:input-FLAG

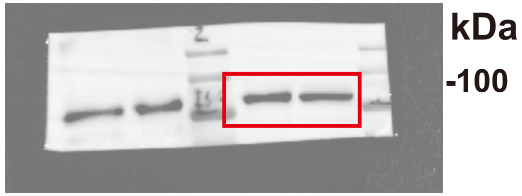

Suppl Fig. 4k:input-β-Actin

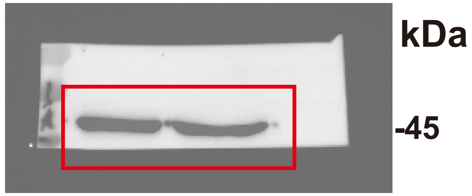

Suppl Fig. 4l:IP-FLAG

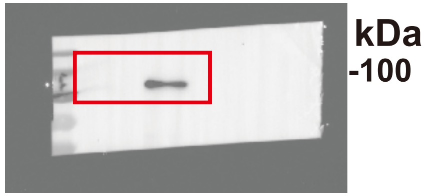

Suppl Fig. 4l:IP-HA

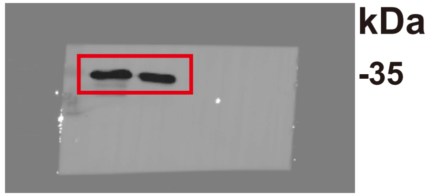

Suppl Fig. 4l:input-FLAG

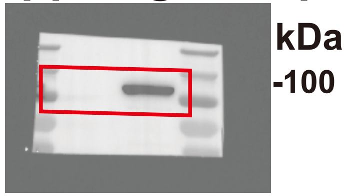

Suppl Fig. 4l:input-HA

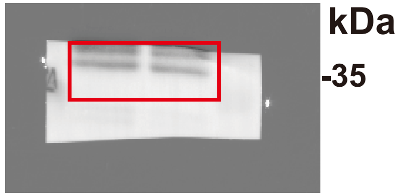

Suppl Fig. 4l:input-β-Actin

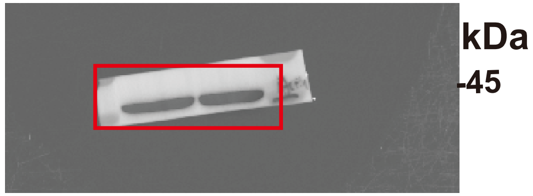

Suppl Fig. 4m:IP-FLAG

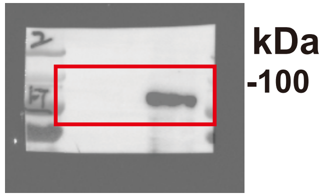

Suppl Fig. 4m:IP-HA

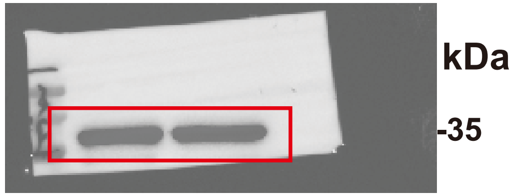

Suppl Fig. 4m:input-FLAG

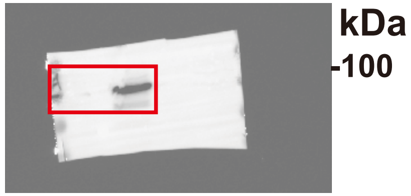

Suppl Fig. 4m:input-HA

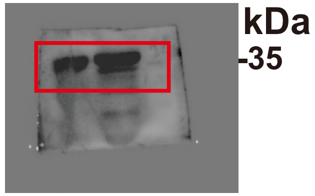

Suppl Fig. 4m:input-β-Actin

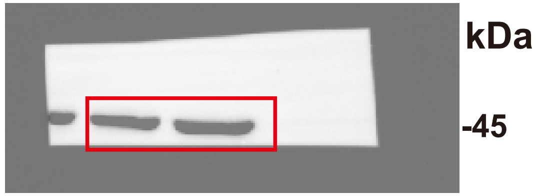

Suppl Fig. 4n:VCP

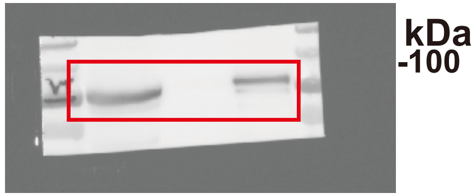

Suppl Fig. 4n:GPD1L

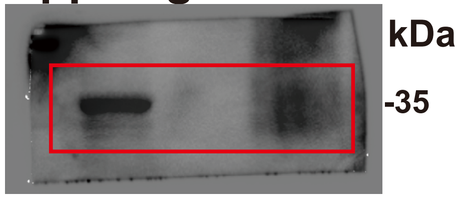

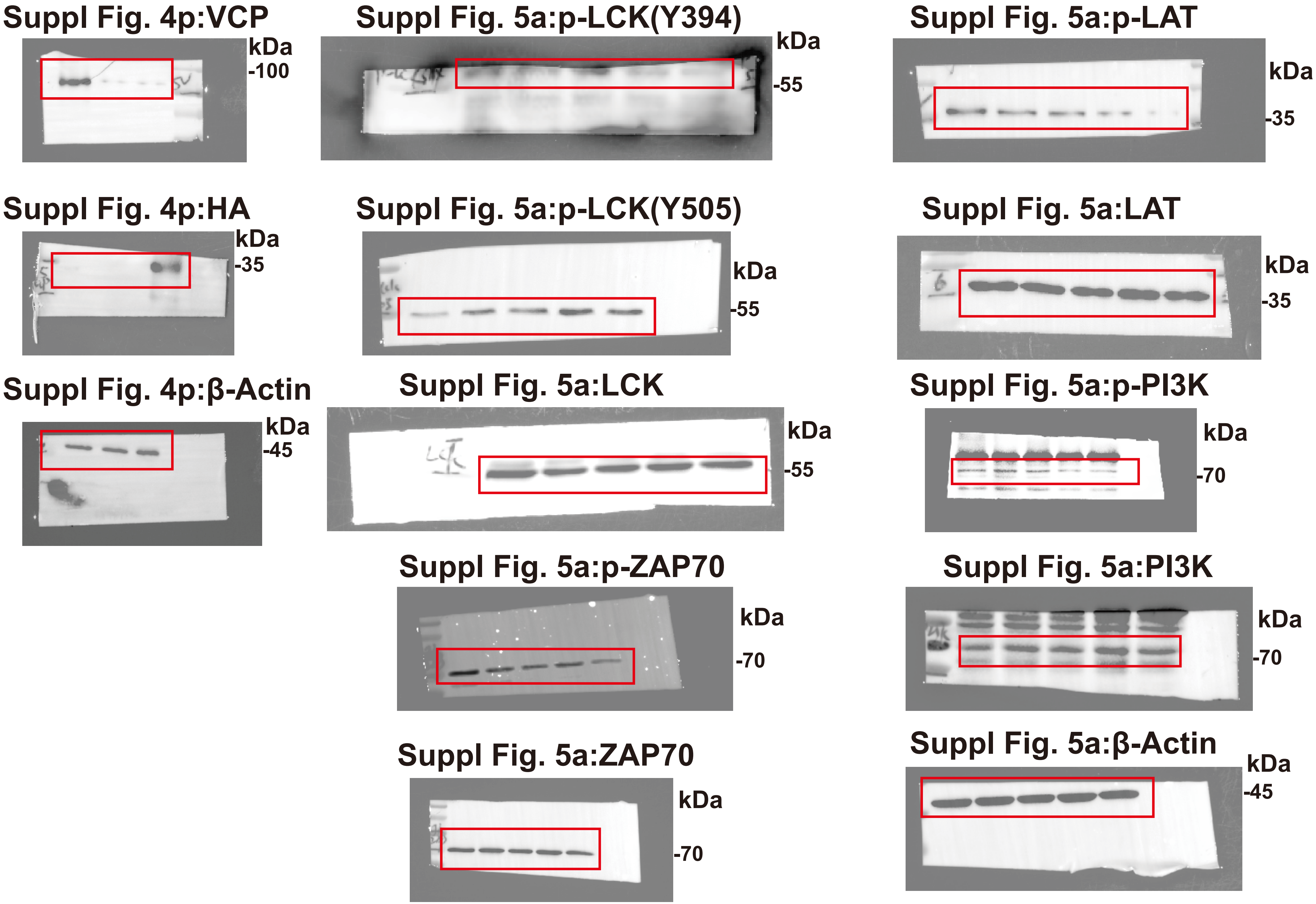
Suppl Fig. 4p:VCP
kDa
-100
Suppl Fig. 5a:p-LCK(Y394)
kDa
-55
Suppl Fig. 5a:p-LAT
kDa
-35
Suppl Fig. 4p:HA
kDa
-35
Suppl Fig. 5a:p-LCK(Y505)
kDa
-55
Suppl Fig. 5a:LAT
kDa
-35
Suppl Fig. 4p:β-Actin
kDa
-45
Suppl Fig. 5a:LCK
kDa
-55
Suppl Fig. 5a:p-PI3K
kDa
-70
Suppl Fig. 5a:p-ZAP70
kDa
-70
Suppl Fig. 5a:PI3K
kDa
-70
Suppl Fig. 5a:ZAP70
kDa
-70
Suppl Fig. 5a:β-Actin
kDa
-45

Suppl Fig. 6b:purified LCK(Y394)

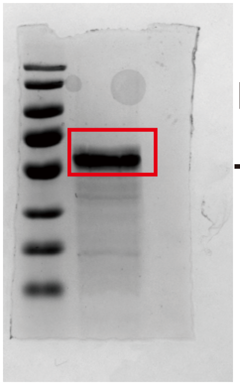

Suppl Fig. 7i:Vcp$^{flox/flox}$

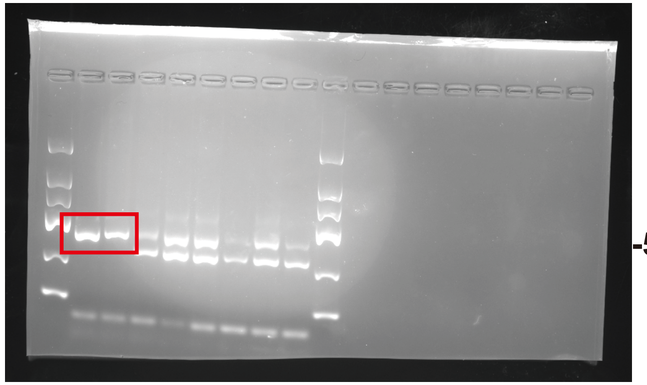

Suppl Fig. 6b:purified LCK(Y505)

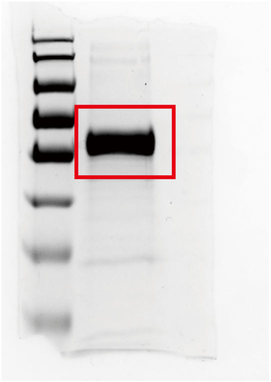

Suppl Fig. 7i:Alb-Cre

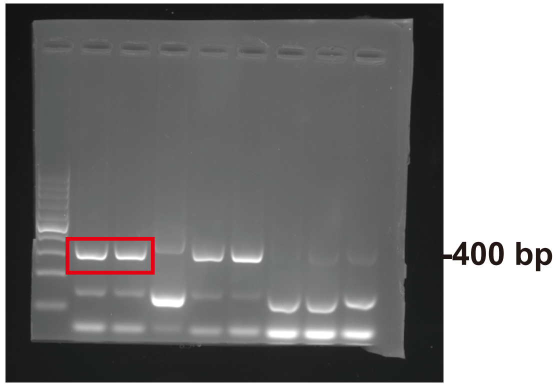

Supplement: Supplementary file 2 — Original WB [file 41392_2024_2120_MOESM2_ESM.pdf]
